# Systematic Review and Meta‐Analysis of Mortality in Patients With Anorexia Nervosa

**DOI:** 10.1111/eat.70002

**Published:** 2025-11-23

**Authors:** Eric Tsz‐Him Lai, Benjamin Lai, Corine Sau‐Man Wong, Lai‐Yi Wong, Kin‐Shing Cheng, Pak‐Wing Calvin Cheng, Lo Heidi Ka‐Ying, Gary Tse, Wai‐Chi Chan, Wing‐Chung Chang, Ka‐Fai Chung

**Affiliations:** ^1^ Department of Psychiatry, LKS Faculty of Medicine The University of Hong Kong Hong Kong SAR China; ^2^ Private Psychiatrist Hong Kong SAR China; ^3^ School of Public Health, LKS Faculty of Medicine The University of Hong Kong Hong Kong SAR China; ^4^ Kwai Chung Hospital Hong Kong SAR China; ^5^ School of Nursing and Health Sciences Hong Kong Metropolitan University Hong Kong SAR China

**Keywords:** anorexia nervosa, death, eating disorders, mortality, standardized mortality ratio, suicide

## Abstract

**Objective:**

Anorexia nervosa (AN) has one of the highest mortality rates among psychiatric disorders. This systematic review and meta‐analysis examined the all‐cause mortality of AN patients compared to the general population using the standardized mortality ratio (SMR).

**Method:**

MEDLINE, PsycINFO, EMBASE, WOS, Dissertations and Theses A&I, and Google Scholar were searched from inception to May 2025 for longitudinal studies reporting all‐cause SMR for AN patients. Risk of bias was assessed using the Newcastle‐Ottawa Scale. Random‐effects meta‐analysis was conducted and presented as a forest plot. Subgroup and meta‐regression analyses were done. SMRs for male‐ and female‐specific samples were compared.

**Results:**

Thirty studies involving 33,176 patients were identified. The pooled SMR from 22 studies was 5.06 (95% CI [3.47–7.38]). Suicide and cardiac deaths accounted for 21% and 19% of deaths, respectively. Studies with lower mean BMI were associated with higher SMRs before correction for multiple testing (*p* = 0.018, adjusted *p* = 0.252). The pooled SMR of male‐specific samples was 3.47, 95% CI (1.60–7.52), similar to female‐specific samples (3.86, 95% CI [1.82–8.20]).

**Discussions:**

Our findings confirm that AN remains a severe psychiatric disorder, underscoring the clinical importance of suicide prevention and monitoring cardiac complications. A low BMI is a crucial clinical indicator for high‐risk groups and allocating resources. Limitations include excluding studies with zero deaths, substantial heterogeneity among included studies, the underrepresentation of male and non‐Western populations, and most studies originating from specialist clinics.

**Key Points**

- Patients with anorexia nervosa (AN) have a standardized mortality ratio (SMR) of 5.06.
- Suicide and cardiac deaths accounted for 21% and 19% of deaths, respectively.
- Studies on patients with lower body mass index (BMI) have shown higher mortality.
- Male and female patients with AN have similar SMR.

## 1 Background

Anorexia nervosa (AN) is recognized as a mental disorder with one of the highest mortality rates (Chesney et al. 2014). A previous widely cited meta‐analysis by Arcelus et al. (2011) estimated the all‐cause mortality rate in AN at 5.10 deaths per 1000 person‐years. Meta‐regression analysis suggested that older age at assessment was associated with higher mortality; however, no significant relationship with body mass index (BMI) was identified, despite BMI being a widely accepted indicator of severity for AN (American Psychiatric Association 2022). Arcelus' meta‐analysis was re‐examined by Keshaviah et al. (2014), who highlighted several methodological concerns, including varying definitions of at‐risk population and duplicated cohorts.

The mortality measurement in AN has evolved from crude mortality proportion to standardized mortality ratio (SMR), which is a ratio of observed to expected deaths based on population mortality statistics, as recommended by a widely cited systematic review (Sullivan 1995). The use of SMR improved comparability across studies, by accounting for variation in follow‐up length and the basal mortality rate of the age‐ and sex‐matched general population. SMR was used as an effect size measurement in the meta‐analysis for all‐cause mortality in eating disorders and other mental disorders (Arcelus et al. 2011; Chesney et al. 2014; Keshaviah et al. 2014). Although there are studies reporting hazard ratio (HR) as the outcome measure of mortality (Auger et al. 2025; Demmler et al. 2020; Duriez et al. 2023; Larsen et al. 2024; Mellentin et al. 2022; Søeby et al. 2024a; Tseng et al. 2023; Zerwas et al. 2015), HR compares the instantaneous risk of events at a particular time with a control group and is therefore conceptually different and not equivalent (Tierney et al. 2025). To summarize the mortality risk of AN compared to the general population, this systematic review focuses on studies that use SMR as the outcome measure.

In the past decade, a growing number of cohort studies have reported inconsistent mortality data in AN, with SMRs ranging from 2.49 to 15.9 (Amemiya et al. 2012; Castellini et al. 2023; Fichter and Quadflieg 2016; Franko et al. 2013; Gueguen et al. 2012; Guinhut et al. 2021; Himmerich et al. 2019; Hoang et al. 2014; Huas et al. 2011; Kask et al. 2016, 2017; Nielsen and Vilmar 2021; Peters et al. 2021; Rosling et al. 2011; Speranza et al. 2019, 2020; Stheneur et al. 2017; Winkler et al. 2015). Some are cohort data from specialized eating disorder service centers which could provide detailed clinical data such as BMI or duration of illness (Fichter and Quadflieg 2016; Franko et al. 2013; Gueguen et al. 2012; Huas et al. 2011; Winkler et al. 2015), while some are based on nationwide health registers which provide large sample sizes, long durations of follow‐up and potentially capture AN patients who are not under specialized care (Himmerich et al. 2019; Hoang et al. 2014; Kask et al. 2016, 2017; Nielsen and Vilmar 2021). Moreover, there is accumulating data on male patients with AN (Kask et al. 2017). This growing body of studies on AN mortality, with diverse methodologies and inconsistent results, has created uncertainty about the true mortality risk in AN.

A recently published meta‐analysis, synthesizing literature from 2010 to 2024, reported an overall SMR of 5.21 for AN based on 25 studies (Krug et al. 2025). While this study provides valuable updated estimates, several methodological aspects warrant further attention. Firstly, the meta‐analysis included a study using Swedish registry data for AN diagnosed from 1973 to 2003 (Papadopoulos et al. 2013), instead of a more recent study with data up to 2010 (Kask et al. 2016). Secondly, it included Quadflieg's study (Quadflieg et al. 2024), which is a subset of 179 adolescent girls also reported in Fichter's study (Fichter and Quadflieg 2016), thereby violating the assumption of study independence in meta‐analysis (Bracken 2016). Thirdly, the choice of outcome measures for the meta‐analysis was not well‐defined, with four studies reporting HRs as outcomes being treated as SMRs in the pooled analysis (Demmler et al. 2020; Larsen et al. 2024; Søeby et al. 2024; Tseng et al. 2023), while two studies using HRs were excluded (Duriez et al. 2023; Suokas et al. 2013). Furthermore, it included studies that did not use formal diagnostic criteria for AN (Crow et al. 2012; Ward et al. 2015). Lastly, it excluded studies published before 2010, which introduced inclusion bias toward studies with a long duration of follow‐up. Therefore an updated meta‐analysis to address these limitations is warranted.

The SMR of all‐cause mortality in patients with AN has been highly variable in the literature. This variability was attributed to methodological factors, such as length of follow‐up, sample size, mortality ascertainment rates, diagnostic criteria, and clinical characteristics of the sample, including age, treatment setting, disorder severity, and psychiatric comorbidities (Jagielska and Kacperska 2017; Kask et al. 2016; Welch and Ghaderi 2015). This variability in mortality has resulted in the high heterogeneity (*I* ^2^ > 90%) detected in previous meta‐analyses (Arcelus et al. 2011; Krug et al. 2025). However, the source of heterogeneity was not adequately examined due to the inadequate extraction of study‐level factors that might modulate the mortality risk. Therefore, this study conducted extensive pre‐specified subgroup and meta‐regression analyses, using moderators from previous reviews, to explore the source of heterogeneity in the literature.

Another uncertainty in the literature is the mortality rate for males with AN, with a systematic review reporting inconclusive results (Strobel et al. 2019) and a more recent review suggesting higher mortality (Iwajomo et al. 2021; van Eeden et al. 2021). Therefore, with an increasing number of studies, further subgroup and meta‐regression analyses can be conducted to explore the study characteristics that affect the variability in reported SMR, as well as an updated understanding of the differences in mortality between males and females.

The understanding of the specific causes of raised mortality in anorexia nervosa has gathered over the years. Previous meta‐analysis showed that 1 in 5 individuals with AN died from suicide (Arcelus et al. 2011). Natural causes of death were also reported to be raised, with cardiac death being the leading cause (Mehler et al. 2022; Søeby et al. 2024). With the accumulating number of studies reporting specific causes of death (Amemiya et al. 2012; Birmingham et al. 2005; Button et al. 2010; Castellini et al. 2023; Fichter and Quadflieg 2016; Franko et al. 2013; Guinhut et al. 2021; Korndorfer et al. 2003; Lee et al. 2003; Lowe et al. 2001; Millar et al. 2005; Rosling et al. 2011; Signorini et al. 2007; Speranza et al. 2019; Stheneur et al. 2017), synthesizing the proportion of deaths due to specific causes can guide our understanding of the mortality of AN.

The objective of this study was to systematically review the current evidence on all‐cause mortality for patients with AN compared to that for the general population, using SMR as the outcome. Other objectives were to investigate the study characteristics (i.e., methodological and clinical) that contributed to variations in SMR using subgroup and meta‐regression analysis, to synthesize the proportion of cause‐specific death, and to explore the difference in SMR between males and females.

## 2 Method

### 2.1 Protocol Registration and Reporting

The protocol of this meta‐analysis was registered with the International Prospective Register of Systematic Reviews (PROSPERO CRD42022329796). The reporting of this systematic review followed the standards of the preferred reporting items for systematic review and meta‐analysis (PRISMA) statement (Page et al. 2021).

### 2.2 Eligibility Criteria

We included studies that (1) diagnosed AN as assessed by criteria as defined by the International Classification of Diseases (ICD‐8, ICD‐9, ICD‐10 or ICD‐11) or the Diagnostic and Statistical Manual of Mental Disorders (DSM‐III, DSM‐III‐R, DSM‐IV, DSM‐IV‐TR, DSM‐5 or DSM‐5‐TR); (2) were cohort studies, randomized controlled trials (RCTs) or follow‐up of RCTs with mortality as an outcome; (3) were written in the English language; (4) followed up patients for at least 1 year; and (5) had 15 or more participants. The exclusion criteria included studies (1) not reporting all‐cause mortality; (2) with missing data that could not be clarified by contacting the author; and (3) with no report of SMR and that could not be derived through available data.

The sample size requirement of 15 or more participants was adopted from Arcelus et al. (2011) to exclude case series that lack objectively defined inclusion criteria.

### 2.3 Developing Search Strategies

We searched six databases, which are MEDLINE (via OVID), PsycINFO (via ProQuest), EMBASE (via OVID), Web of Science Core Collection, Dissertations and Theses A&I databases (via ProQuest), and Google Scholar. The search was done from inception to 1 April 2023, with an updated search up to 1 May 2025. For Google Scholar, the first 200 articles were included (Bramer et al. 2017). Search terms related to the disease name, that is, “eating disorders” or “anorexia nervosa”, were combined with terms associated with the outcome of interest, that is, “mortality”, “fatality”, “survival”, or “death”, using the Boolean operator “AND”. The opposite meaning of “mortality”, that is, “survival”, was searched to avoid bias. Forward and backward citation searches were done using the Web of Science core collection on 16 May 2023. Forward citation search used the included studies, while backward citation search used the included studies and relevant review articles found during the search process. Full search strategies are provided as Supporting Information.

### 2.4 Study Selection

The search results from different databases were imported into Rayyan (Ouzzani et al. 2016). Duplicated studies were flagged by the application and manually deleted by E.T.H.L. The selection of relevant publications was carried out independently by two reviewers (E.T.H.L. and B.L.) based on the eligibility criteria. Titles and abstracts were all double‐screened to remove irrelevant reports. E.T.H.L. retrieved full texts, and all were double‐screened. Any disagreements were resolved by consensus with K.S.C.

The author's name, study period, and data collection site were used to detect multiple reports of the same dataset or overlapping data. Studies with duplicated mortality outcome data or data sets from overlapping periods at the same site were identified. The authors were approached for clarification when needed. The studies with the longest follow‐up duration were chosen for inclusion in the systematic review. The authors of potentially eligible studies that fit the inclusion and exclusion criteria were contacted to request further information and inquire if they were aware of any relevant unpublished studies.

### 2.5 Data Extraction

Two reviewers (E.T.H.L. and B.L.) extracted data independently using a pre‐established form in Google Sheets (Supporting Information). Disagreements were resolved through consensus. The data were obtained directly from the original articles. The authors were contacted for additional information when needed.

While the mean BMI for the cohort reported in Eckert et al. (1995) was not reported in the study, the individual patient‐level data were reported in Halmi et al. (1979), which allowed an estimation of the mean BMI to be 13.8 kg/m^2^. This is consistent with the description of the cohort's mean weight being 31.1% below normal (Eckert et al. 1995).

### 2.6 Quality Assessment

The Newcastle‐Ottawa Quality Assessment Scale (NOS) for cohort study was used for quality assessment (Wells et al. 2000). The studies were independently rated by two reviewers (E.T.H.L. and B.L.), with differences resolved through consensus. The wording of NOS was adapted for this systematic review (Supporting Information). The study quality was graded as good, fair, or poor according to the Agency for Healthcare Research and Quality (AHRQ) cut‐off (Shamsrizi et al. 2020).

### 2.7 Effect Measures

The SMR was chosen as the effect measure. The SMR was the observed number of deaths divided by the expected number of deaths. An SMR greater than 1 meant that the mortality rate of AN was greater than that of the age and sex‐matched general population. When a paper reported the number of deaths but not the SMR, the author was contacted for raw data for computing the SMR. The 95% confidence intervals (CI) were extracted from the studies or calculated if they were not provided.

### 2.8 Data Analysis

The SMR was log‐transformed for meta‐analysis. Due to the expected heterogeneity across studies, the random effects model was used. The restricted maximum likelihood estimator was adopted with Knapp–Hartung adjustments for the CI (Knapp and Hartung 2003; Langan et al. 2019; Viechtbauer 2005). A forest plot was used to visually display the results. The heterogeneity between studies was evaluated using Cochran's Q statistic, with a *p*‐value less than 0.10 indicating significant heterogeneity. The *I*‐squared statistic was computed, where 0%, 25%, 50%, and 75% indicate no, low, moderate, and high heterogeneity, respectively (Higgins et al. 2003). All statistical analyses were performed within the R programming environment (R Foundation for Statistical Computing). The packages for meta‐analysis, namely meta, metafor, and dmeta, were used. The 95% CI for the SMR was calculated using the epi.smr function from the package epiR when it was not available from the paper. For studies with observed deaths of five or fewer, the mid‐p exact test was used. Otherwise, the Byar approximation was used. Graphing was performed using the package ggplot2. The certainty of evidence was evaluated using the GRADE approach (Supporting Information).

To further understand the specific cause of mortality, the proportions of deaths by specific causes were pooled using a generalized linear mixed‐effect model (GLMM), which was recommended for meta‐analysis of proportions (Schwarzer et al. 2019). Only studies with less than 10% unknown causes of death were included in the analysis.

The publication bias was visualized with a funnel plot and evaluated by Egger's test. Studies with CI not overlapping with the CI of the pooled effect were identified as outliers. The influence analyses were done by the leave‐one‐out method. The analyses were done using the dmeta package (Harrer et al. 2021). Sensitivity analyses were conducted to investigate the impact of alternative combinations of selecting studies.

### 2.9 Sub‐Group and Meta‐Regression Analysis

In view of the expected heterogeneity among included studies based on previous meta‐analyses, a list of pre‐specified study characteristics that could affect SMR was investigated using subgroup and meta‐regression analyses. The list of study characteristics of interest was derived from two reviews on the predictors of mortality in AN and hypothesized that study‐level summaries of these moderators could predict the SMR (Jagielska and Kacperska 2017; Welch and Ghaderi 2015). The mortality of various subgroups was examined using subgroup analysis. The subgroups included different treatment settings, different diagnostic criteria, gender, and different study qualities. There was one post hoc addition of subgroup analyses after observing two distinct groups of studies included, namely population‐based and clinical‐based studies. The population‐based studies were those that aimed to sample patients from the whole population, usually from health registries (Himmerich et al. 2019; Hoang et al. 2014; Kask et al. 2016, 2017; Nielsen and Vilmar 2021) or from all healthcare providers in an area (Korndorfer et al. 2003), leading to a sample representative of those with anorexia nervosa in the general community. While clinical‐based studies sampled patients from psychiatry or eating disorders specialist units. Power analysis was done for the subgroup analysis of patient gender (Hedges and Pigott 2001).

The influence of various sample characteristics with continuous values was studied using bivariate meta‐regression analysis. These included the mean age, mean BMI at study entry, length of follow‐up, sample size, ascertainment rate, percentage of patients with psychiatric comorbidities, and percentage of patients with binge‐purging subtype. In response to peer review, we conducted further exploratory meta‐regression analyses to investigate sources of heterogeneity, particularly potential temporal changes in the mortality of patients with AN. Our primary hypothesis was that SMRs may have decreased in more recent decades due to advancements in care, which we tested using the midpoint of each study's recruitment period as a moderator. However, we anticipated a potential correlation between recruitment year and the overall length of a study, which could confound this relationship. To represent this methodological factor, we used the total duration of study follow‐up, calculated as the time in years from the start of recruitment to the date of final mortality ascertainment. To disentangle the effect of the care era from this methodological influence of study duration, both variables were assessed individually and then included in a multivariable meta‐regression model to estimate their independent effects. We required at least five studies for each independent variable to be included in meta‐regression (van Houwelingen et al. 2002). Individual *p*‐values of each test were reported for exploring potential moderators, while adjusted *p*‐values using Bonferroni corrections were also reported to account for the 5 subgroups and 9 meta‐regression analyses (Rothman 1990).

## 3 Result

### 3.1 Study Selection

**FIGURE 1:**
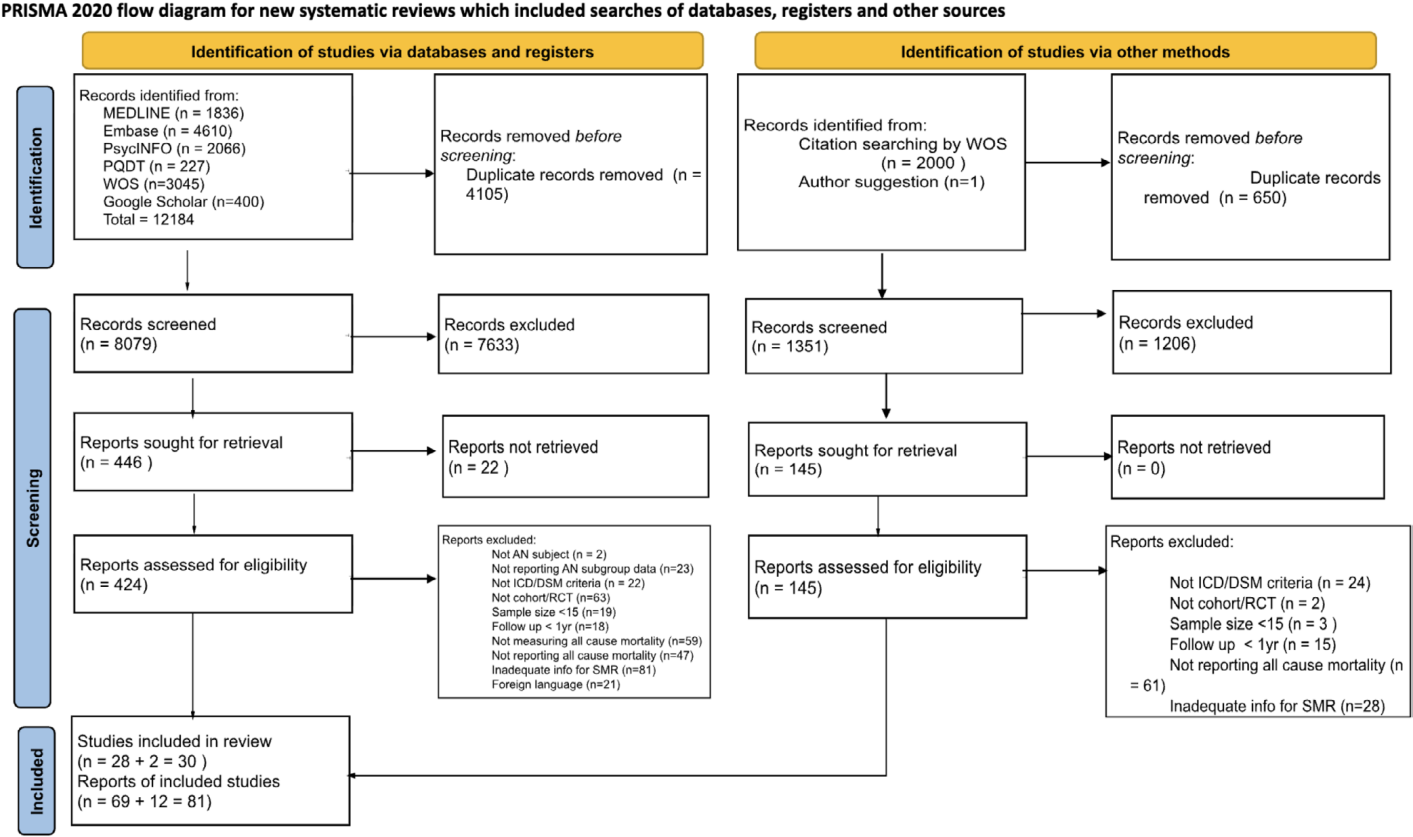
PRISMA 2020 flow diagram for systematic reviews. This is a PRISMA flow diagram showing the study selection process. AN, anorexia nervosa; DSM, Diagnostic and Statistical Manual of Mental Disorders; ICD, International Classification of Diseases; PQDT, Dissertations and Theses A&I databases (via ProQuest); RCT, randomized controlled trial; WOS, Web of Science. *Source*: Page MJ, McKenzie JE, Bossuyt PM, Boutron I, Hoffmann TC, Mulrow CD, et al. The PRISMA 2020 statement: An updated guideline for reporting systematic reviews. BMJ 2021; 372: N71. https://doi.org/10.1136/bmj.n71 .

The study selection process is shown in Figure 1. The search of the electronic database resulted in 12,184 records. Manual de‐duplication removed 4105 records. The titles and abstracts of 8079 records were screened, and 7633 were excluded based on the eligibility criteria. There were 446 reports successfully retrieved, and 69 of them were eligible. After carefully comparing and removing multiple reports of the same study, we selected 28 studies that met our criteria. Multiple reports of selected studies, overlapping studies, and a list of non‐English studies excluded are presented as Supporting Information.

Based on the 28 studies and 13 reviews retrieved from the search process, forward and backward citation searches were performed, and 2000 additional records were found. There were 12 eligible reports, and 10 were from studies already included. Therefore, two additional studies were included, resulting in a total of 30 cohort studies. We initiated 92 contacts with authors via email and received 34 responses (response rate = 37%).

A clinical follow‐up study by Rosling et al. from Sweden was overlapped by a large national patient register data study by Kask et al. (Kask et al. 2016, 2017; Rosling et al. 2011). Three clinical follow‐up studies from Denmark by Joergensen (1992), Pagsberg and Wang (1994) and Winkler et al. (2015) overlapped with the national patient register data study by Nielsen and Vilmar (2021). Twenty‐seven patients within the 157 male AN patients in Quadflieg et al. (2019) were previously reported by Fichter and Quadflieg (2016), which had a mixed‐gender sample of 1693 AN patients. Both studies were retained in our systematic review as they were invaluable for understanding the gender differences in mortality.

### 3.2 Study Characteristics

The characteristics of the selected studies are summarized in Table 1. All 30 included studies were cohort studies published between 1994 and 2023. The sample size ranged from 25 to 9143, with a median of 251.5. The follow‐up durations ranged from 1 year to 27.1 years, with a median of 7.4 years. There were 23 European studies, five from North America and two from Asia. Five of the 30 studies were registry studies, analyzing administrative health data instead of clinical data from clinical records or interviews. Four of the five registry studies were nationwide studies from Denmark, Sweden and England (Hoang et al. 2014; Kask et al. 2016, 2017; Nielsen and Vilmar 2021), while one study used case registers of Southeast London (Himmerich et al. 2019). Most of the clinical studies were performed at specialist centres, except for Korndorfer et al., which is a community‐based study that recruited all 208 Rochester residents who first presented with AN (Korndorfer et al. 2003). The studies used various diagnostic criteria: DSM‐IV in 13 studies, ICD‐10 (F50.0, F50.1) in four studies, DSM‐III‐R in four studies, and DSM‐III and DSM‐5 each in one study. Seven studies employed a mixture of diagnostic criteria.

**TABLE 1:** Characteristics of selected studies examining the standardized mortality ratio (SMR) in patients with anorexia nervosa.

Author (year)	Country	Recruitment setting	Sample size recruited (n0)/with known vital status (n)/mean follow‐up (FU)	Diagnostic criteria	Assessment method	Mean BMI	Mean age	Male %	Race/ethnicity	Socioeconomic status	% of binge purging	Psychiatric comorbidity %	SMR (95% CI)
Castellini et al. (2023)	Italy	Attendee of regional treatment centres for ED in 1994–2018 Outpatient/inpatient/day hospital	*n* = 368, FU = 6.1 years	DSM‐5	SCID	16.5	25.6	2	NR	NR	45	—	2.5 (0.8–7.7)
Peters et al. (2021)	Germany	Cases discharged from an inpatient ED unit in 2016–2019	*n*0 = 304, *n* = 291, FU = 6.0 years	ICD‐10 (F50.0 F50.1)	Clinical interview	15.6	26.4	0	NR	NR	38	67.1	4.7 (0.2–23.1)
Nielsen and Vilmar (2021)	Denmark	All patients appeared in the danish central psychiatric research register with an ED diagnosis in 1970–2014	*n*0 = 9143, *n* = 9143, FU = 12.6 years	ICD‐8,10 (F50.0, F50.1)	Registry diagnostic code	—	20.7	6	NR	NR	—	—	3.4
Guinhut et al. (2021)	France	Patients first admitted to specialized inpatient medical unit in 1997–2014	*n*0 = 386, *n* = 384, FU = 5.2 years	DSM‐IV	Clinical interview	12.7	29.4	6	NR	25.3% with ≥ 4 years of higher education	48	48.2	15.9 (11.6–21.4)
Himmerich et al. (2019)	UK	All patients received AN diagnosis from a regional ED tertiary service within or before 2007–2016	*n*0 = 1970, *n* = 1970	ICD‐10 (F50.0 F50.1)	Registry diagnostic code	—	24.5	7	83.2% white, 3.20% Black, 13.6% others	Stratified by IMD score tertiles^a^	—	—	5.2 (3.8–7.0)
Speranza et al. (2020)	Italy	Patients who attended the outpatient ED unit from 2000 to 2005 were reviewed in 2017	*n*0 = 117, *n* = 117	DSM‐IV	Clinical interview	16.1	20.0	0	NR	NR	0	—	6.9
Quadflieg et al. (2019)	Germany	Patients treated in an ED inpatient unit from 1985 to 2017	*n*0 = 157, *n* = 147, FU = 6.6 years	DSM‐IV	Standardized algorithm of a self‐rating scale	15.4	27.3	100	NR	NR	66	—	5.9 (3.6–9.2)
Kask et al. (2017)	Sweden	Inpatient male aged 10–40 discharged with AN as a main or second diagnosis on the registry from 1973 to 2010	*n*0 = 609, *n* = 609	ICD‐8,9,10 (F50.0, F50.1)	Registry diagnostic code	—	18.2	100	NR	NR	—	42.0	4.1 (3.1–5.3)
Stheneur et al. (2017)	France	Consecutively admitted AN patients from 1996 to 2002 to a psychiatric unit. Vital status collected in 2008	*n*0 = 180, *n* = 180	DSM‐IV	Clinical interview	13.3	17.1	0	NR	NR	—	—	6.1
Fichter and Quadflieg (2016)	Germany	Inpatients admitted to ED unit from 1985 to 2005. Follow‐up data collected from 2007 to 2012	*n*0 = 1693, *n* = 1639	DSM‐IV	Standardized algorithm of a self‐rating scale	14.8	25.0	2	NR	NR	69	56.0	5.4 (4.3–6.5)
Kask et al. (2016)	Sweden	Inpatient female aged 10–40 discharged with AN as a main or second diagnosis on the registry from 1973 to 2010	*n*0 = 8098, *n* = 8069, FU = 15.3 years	ICD‐8,9,10 (F50.0, F50.1)	Registry diagnostic code	—	19.5	0	NR	NR	—	41.9	5.2 (4.7–5.7)
Winkler et al. (2015)	Denmark	Patients were referred to an ED centre from 1994 to 2004. Vital status obtained in 2011	*n*0 = 396, *n* = 396	DSM‐IV	Clinical interview and note review	—	—	—	NR	NR	—	—	2.9 (1.6–5.1)
Hoang et al. (2014)	UK	NHS inpatients discharged with AN as the primary diagnosis from 2001 to 2009. Death within 1 year from discharge was recorded.	*n*0 = 5880, *n* = 5880, FU = 1 year	ICD‐10 (F50.0 F50.1)	Registry diagnostic code	—	—	8	NR	NR	—	—	3.4 (2.2–4.6)
Franko et al. (2013)	US	Outpatients recruited to a longitudinal ED study from 1987 to 1991. Vital status obtained in 2010	*n*0 = 186, *n* = 186, FU = 20.0 years	DSM‐IV	Semi‐structured interview	18.8	24.5	0	NR	NR	63	—	4.4 (2.4–7.3)
Amemiya et al. (2012)	Japan	Inpatients admitted to a psychiatric unit from 1997 to 2002. Patients were follow‐up 2006–2007.	*n*0 = 88, *n* = 67, FU = 6.3 years	DSM‐IV	SCID	13.4	21.6	0	Japanese	NR	57	—	2.0 (0.5–5.1)
Gueguen et al. (2012)	France	Male inpatients who were first admitted to an ED unit from 1988 to 2004. Vital status was collected in 2008.	*n*0 = 23, *n* = 23, FU = 9.8 years	DSM‐IV	Clinical interview and notes review	15.6	26.6	100	NR	65.2% with higher education	61	—	8.1 (1.6–23.6)
Huas et al. (2011)	France	Female inpatients who were first admitted to an ED unit from 1988 to 2004. Vital status was collected in 2008.	*n*0 = 601, *n* = 539, FU = 10.0 years	DSM‐IV	Clinical interview and notes review	14.5	26.4	0	NR	64.2% with higher education	47	—	10.6 (7.6–14.4)
Rosling et al. (2011)	Sweden	Inpatient admitted to an ED unit from 1974 to 1993. Vital status determined in 2000	*n*0 = 157, *n* = 157	DSM‐IV	Clinical interview and notes review	—	—	0	NR	NR	—	93.0	11.7 (7.2–17.8)
Button et al. (2010)	UK	Patients assessed by an ED service from 1992 to 2004. Vital status was determined in 2007.	*n*0 = 295, *n* = 295	DSM‐III‐R, DSM‐IV	Clinical interview	—	—	0	NR	NR	—	—	9.8 (4.7–18)
Signorini et al. (2007)	Italy	Outpatient attended an ED unit from 1994 to 1997. Patient information collected in 2003	*n*0 = 147, *n* = 138, FU = 8.0 years	DSM‐IV	Clinical interview	16.2	20.5	0	NR	NR	0	—	9.7
Crisp and Collaborators (2006)	UK	A data set was collected by Crisp from 1960 to 1967 while working in a medical school, then by Crisp's team in St Geoge's Hospital to 1990.	*n*0 = 935, *n* = 935	DSM‐IV (AN, hx AN)	Clinical interview	—	—	9	NR	NR	—	—	1.0
Millar et al. (2005)	UK	All inpatients, day patients or outpatients seen in regional specialist services from 1965 to 1999. Vital status determined up to 2002	*n*0 = 524, *n* = 507	ICD‐9, 10 (F50.0)	Clinical interview and notes review	16.6	20.1	7	NR	NR	—	—	3.3 (2.2–4.9)
Birmingham et al. (2005)	Canada	Outpatients referred to ED unit 1981–2000.	*n*0 = 326, FU = 7.3 years	DSM‐III, DSM‐III‐R, DSM‐IV	Clinical interview	—	24.7	—	NR	NR	—	—	10.5 (5.5–15.5)
Korndorfer et al. (2003)	US	Inpatients and outpatients presented to regional health services for the first time from 1935 to 1989. Vital status updated until 2000.	*n*0 = 208, FU = 27.1 years	DSM‐III‐R	Clinical interview and notes review	—	21.5	7	All patients were white	NR	—	—	0.7 (0.4–1.1)
Lee et al. (2003)	Hong Kong	Patients with an onset of illness at least 4 years seen at an ED clinic from 1984 to 2000.	*n*0 = 88, *n* = 88, FU = 6.7 years	DSM‐III‐R	Clinical interview	14.4	20.4	0	All Chinese	UK Registrar General's social class (paternal occupation)^b^	33	—	10.5
Lowe et al. (2001)	Germany	Inpatients treated from 1971 to 1980, follow‐up from 1997 to 1998.	*n*0 = 84, *n* = 84, FU = 21.3 years	DSM‐IV	Clinical interview	13.3	20.7	0	NR	19% university graduate at 12‐years follow‐up	43	32.0	9.8
Crow et al. (1999)	US	Patients above the age of 25 that a psychiatrist saw in the emergency room from 1985 to 1990.	*n*0 = 25, *n* = 25, FU = 7.5 years	DSM‐III	Clinical interview	—	—	—	NR	NR	—	—	8.8
Eckert et al. (1995)	US	Inpatient participated in a treatment study	*n*0 = 76, *n* = 76, FU = 9.6 years	DSM‐III‐R	Clinical interview	13.8	20.0	0	NR	NR	47	—	12.8
Pagsberg and Wang (1994)	Denmark	Regional inpatients that were hospitalized from 1970 to 1990. Regional patients registered in a mental disorder study from 1970 to 1983. Outpatient attended local psychiatric service. Patients treated in the somatic department. Patients who were known to general practitioners.	*n*0 = 50, *n* = 50, FU = 6.2 years	ICD‐10 (F50.0 F50.1)	Clinical interview and review clinical information	—	21.2	6		NR	NR	—	6.9 (1.4–20.2)
Joergensen (1992)	Denmark	Psychiatric outpatient and inpatient. Somatic department inpatients. From 1977 to 1986. Patients who were known to general practitioners.	*n*0 = 62, *n* = 62, FU = 11.7 years.	DSM‐III‐R	Clinical interview and review hospital records	—	—	8		NR	NR	—	11.2 (6.3–19.7)

*Note*: Unless otherwise specified, the vital status of the cohort was collected in the last year of the recruitment period described in the recruitment setting.

Abbreviations: “—”, unavailable data; ED, eating disorders; NR, not reported; SCID, structured clinical interview for DSM.

^a^ The sample was stratified into three evenly distributed tertiles based on the IMD score: Low (score 1.14–17.60; 32.7%), Medium (17.61–29.17; 32.6%), and High (29.18–100; 32.7%). An additional 1.9% of the sample was reported as missing/homeless.

^b^ Based on the U.K. Registrar General's classification of paternal occupation. The distribution was: Class I (professional): 5.7% (*N* = 5); Class II (managerial/intermediate): 9.1% (*N* = 8); Class III (skilled): 27.3% (*N* = 24); Class IV (partly skilled): 47.7% (*N* = 42); and Class V (unskilled): 10.2% (*N* = 9).

The total number of AN patients included was 33,176. The mean age of the patient samples ranged from 17 to 29.4 years. There were 14 studies recruiting patients from inpatient settings, three studies from outpatient settings (Franko et al. 2013; Signorini et al. 2007; Speranza et al. 2020), and others from a mixture of inpatient and outpatient settings. Three studies reported SMR for male patients (Gueguen et al. 2012; Kask et al. 2017; Strobel et al. 2019), six studies separately reported SMR for males and females (Crisp and Collaborators 2006; Guinhut et al. 2021; Hoang et al. 2014; Korndorfer et al. 2003; Millar et al. 2005; Nielsen and Vilmar 2021), and the other studies included less than 10% of male subjects. The total number of male patients involved was 2143. Six studies stated the percentage of patients with psychiatric comorbidities, which ranged from 32% to 93%. Fourteen studies described the percentage of binge purging subtypes, ranging from 0% to 69%.

One study reported the SMR for males and females separately (Nielsen and Vilmar 2021). Two studies on male AN were published separately from their female patients' data, from Sweden (Kask et al. 2016, 2017) and France (Gueguen et al. 2012; Huas et al. 2011). Including these three data pairs separately for meta‐analysis would violate the assumption of statistical independence of the effect size, artificially reduce heterogeneity, and allocate more weight to the study. Therefore, the SMR and other covariates from the male and female samples of the same study were combined, providing one effect size for each study as a unit, and resulting in 28 study units from 30 studies being synthesized for all‐cause mortality. The male and female SMRs would be analyzed separately when investigating gender differences in subgroup analysis.

All 30 included studies used indirect standardization to compute all‐cause SMR. We calculated the SMR of Peters et al. using raw data provided upon request and Germany's mortality data (Peters et al. 2021; WHO Mortality Database—WHO, n.d.). Two authors published the SMR for the two included studies (Joergensen 1992; Pagsberg and Wang 1994) in a quantitative summary (Nielsen et al. 1998). The SMR for Speranza et al. was extracted from a published conference abstract (Speranza et al. 2019, 2020). All included studies had non‐zero mortality rates. There were 25 studies providing information about the cause of death. In addition to the all‐cause SMR, only eight studies reported cause‐specific SMRs, as summarized in Table 2.

**TABLE 2:** Cause‐specific mortality reported in the included studies.

Studies	All‐cause SMR (95% CI)	Cause‐specific SMR (95% CI)
Castellini et al. (2023)	2.49 (0.8–7.74)	neoplasm 1.71 (0.24–12.13)
Nielsen and Vilmar (2021)	3.38 (3.07–3.72)	suicide 11.0 (7.76–11.2)
Kask et al. (2017)	4.1 (3.1–5.3)	natural 3.9 (2.7–5.4); unnatural 4.5 (2.9–6.6); suicide “Seven‐fold increase”
Kask et al. (2016)	5.2 (4.7–5.7)	natural 4 (3.5–4.6); unnatural 8.5 (7.3–9.9); suicide 13.2 (11.0–15.7)
Hoang et al. (2014)	3.4 (2.2–4.6)	unnatural 1.6 (0–3.9) suicide 2.4 (0–7.2)
Franko et al. (2013)	4.37 (2.4–7.3)	suicide 25.2 (6.9–64.5)
Rosling et al. (2011)	11.7 (7.2–17.8)	suicide 29.3 (13.4–55.5)
Korndorfer et al. (2003)^a^	0.71 (0.42–1.09)	natural 0.57 (0.30–0.97), unnatural 1.36 (0.23–3.96)

^a^ Korndorfer et al. (2003) reported SMR for each ICD‐9‐CM code and were translated to natural and unnatural causes in this table. ICD‐9‐CM code 800–999 was considered unnatural death, ICD‐9‐CM code 780–799 was excluded as ill‐defined, and others were considered natural death.

### 3.3 Study Quality Assessment

Study quality was rated using the NOS with the AHRQ cut‐off, as reported in Table 3. There were 11 studies with good quality, 14 with fair quality and five with poor quality. The inter‐rater reliability of the study quality was found to be high (*κ* = 0.89, *n* = 30). The differences were resolved through consensus.

**TABLE 3:** Newcastle‐Ottawa quality assessment form for selected Cohort studies reported SMR in patients with AN.

	Selection				Comparability	Outcome				
	Representativeness of the exposed cohort	Selection of the non‐exposed cohort	Ascertainment of exposure	Demonstration that outcome of interest was not present at start of study	Comparability of cohorts on the basis of the design or analysis	Assessment of outcome	Was follow‐up long enough for outcomes to occur	Adequacy of follow‐up of cohorts	Total	AHRQ
Castellini et al. (2023)	*	*	*	*	**	*	*		8	Good
Peters et al. (2021)			*	*	**			*	5	Poor
Nielsen and Vilmar (2021)	*	*		*	**	*	*	*	8	Good
Guinhut et al. (2021)			*	*	**	*	*	*	7	Fair
Himmerich et al. (2019)	*		*	*	**	*	*	*	8	Good
Speranza et al. (2019)				*	**	*		*	5	Poor
Franko et al. (2013)	*	*	*	*	**	*	*	*	9	Good
Quadflieg et al. (2019)			*	*	**	*	*	*	7	Fair
Kask et al. (2017)		*		*	**	*	*	*	7	Fair
Stheneur et al. (2017)			*	*	**	*		*	6	Fair
Fichter and Quadflieg (2016 )			*	*	**	*	*	*	7	Fair
Kask et al. (2016)			*	*	**	*	*	*	7	Fair
Winkler et al. (2015)	*		*	*	**	*	*	*	8	Good
Joergensen (1992)	*			*	**		*	*	6	Fair
Hoang et al. (2014)			*	*	**	*	*	*	7	Fair
Amemiya et al. (2012)			*	*	**		*		5	Poor
Gueguen et al. (2012)			*	*	**	*		*	6	Fair
Huas et al. (2011)			*	*	**	*	*		6	Fair
Rosling et al. (2011)			*	*	**	*	*	*	7	Fair
Button et al. (2010)	*		*	*	**	*	*	*	8	Good
Signorini et al. (2007)				*	**			*	4	Poor
Crisp and Collaborators (2006)				*	*	*	*	*	5	Poor
Millar et al. (2005)	*	*	*	*	**	*	*	*	9	Good
Birmingham et al. (2005)	*	*	*	*	**	*	*		8	Good
Korndorfer et al. (2003)	*		*	*	**	*	*	*	8	Good
Lee et al. (2003)	*	*	*	*	**	*		*	8	Good
Lowe et al. (2001)			*	*	**	*	*	*	7	Fair
Crow et al. (1999)			*	*	**	*		*	6	Fair
Eckert et al. (1995)			*	*	**	*	*	*	7	Fair
Pagsberg and Wang (1994)	*		*	*	**		*	*	7	Good

*Note*: The allocation of “*” and “**” is according to the adapted Newcastle‐Ottawa Scale (Supporting Information).

### 3.4 Result Synthesis

**FIGURE 2:**
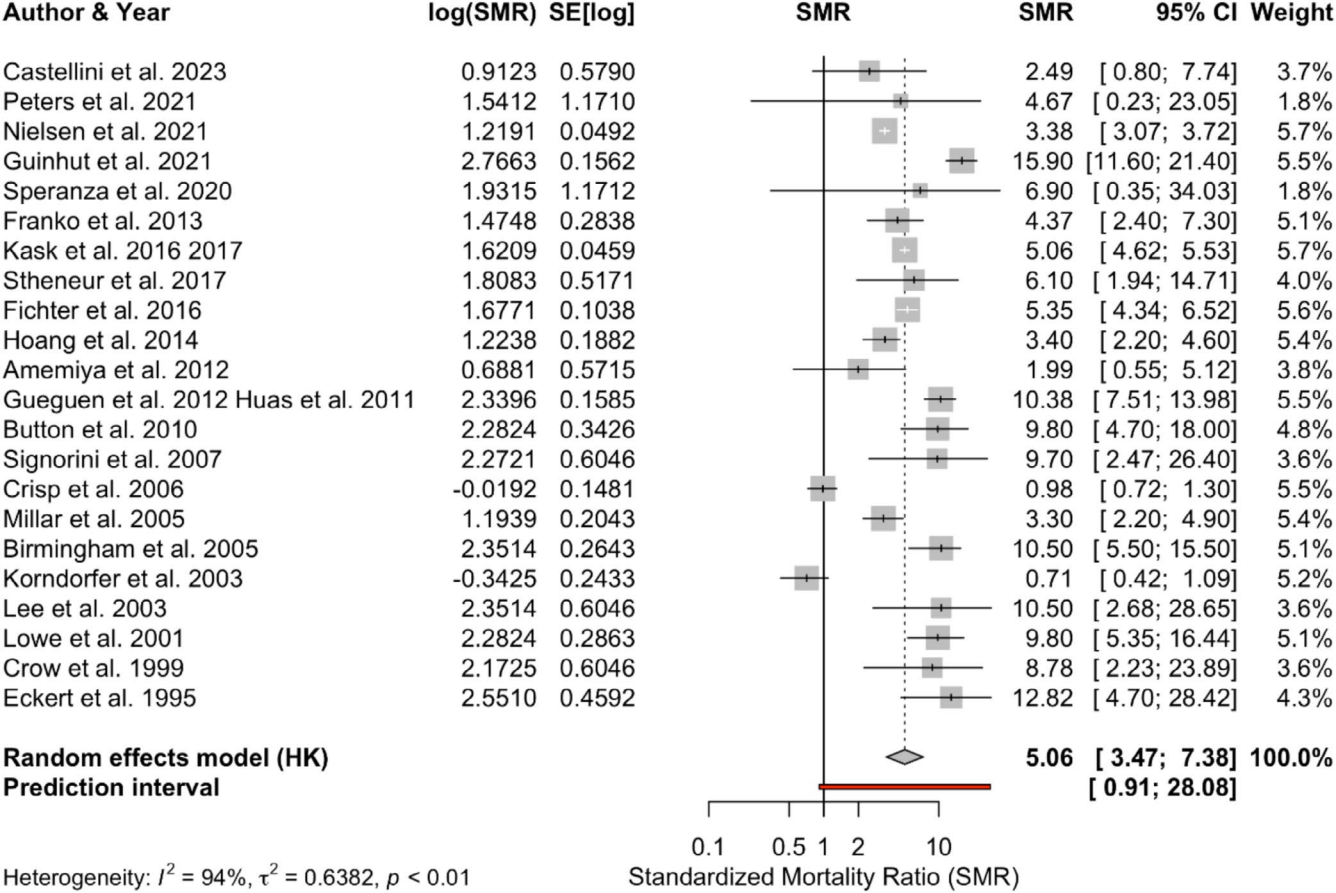
The forest plot of 22 study units for all‐cause SMR in AN. This is the forest plot of the main meta‐analysis, including 22 study units from 30 studies that met the inclusion and exclusion criteria. Two study pairs were combined, that is, Gueguen et al. (2012) and Huas et al. (2011), and Kask et al. (2016) and Kask et al. (2017), as they were male and female samples from the same eating disorder unit, recruited over the same period. Six studies in the systematic review were not included in this main meta‐analysis as their sample overlapped with some included larger studies (Joergensen 1992; Pagsberg and Wang 1994; Rosling et al. 2011; Winkler et al. 2015; Himmerich et al. 2019; Quadflieg et al. 2019). The pooled SMR was 5.06, significantly elevated compared with the general public. The restricted maximum likelihood estimator was used for the between‐study effect size variance. AN, anorexia nervosa; HK, Hartung‐Knapp modification.

In order to avoid overlapping studies and to preserve the studies with most patient data for the primary meta‐analysis, the four smaller studies on clinical samples from Sweden and Denmark (Joergensen 1992; Pagsberg and Wang 1994; Rosling et al. 2011; Winkler et al. 2015) that were being overlapped by the two large study units with data from the Sweden and Denmark patient registers were not included (Kask et al. 2016, 2017; Nielsen and Vilmar 2021). The study by Quadflieg et al. (2019), which overlapped with Fichter and Quadflieg (2016) and had a smaller sample size, was excluded from the primary meta‐analysis. Himmerich et al. (2019) was removed as it was overlapped by Hoang et al. (2014), which has a larger sample size. These exclusions would avoid study overlap and maintain study‐unit independence, while ensuring the inclusion of the largest number of patients. The remaining 22 study units involved 30,384 patients with AN. The pooled SMR was 5.06, with a 95% CI (3.47–7.38), which was significantly elevated compared to the general public. The between‐study heterogeneity variance was estimated at 2 = 0.64, 95% CI (0.30–1.24), with an *I* ^2^ value of 93.8%, signifying high between‐study heterogeneity. The 95% prediction interval was (0.91–28.08). The forest plot is presented in Figure 2.

Of the 22 study units in the meta‐analysis, two studies did not report their natural or unnatural causes of death (Crisp and Collaborators 2006; Hoang et al. 2014), while five study units from six studies had more than 10% of cases of death undefined (Fichter and Quadflieg 2016; Gueguen et al. 2012; Guinhut et al. 2021; Huas et al. 2011; Lowe et al. 2001; Millar et al. 2005). Among the 15 study units that reported the natural and unnatural causes of death, with less than 10% of the causes of death undetermined, there were 994 observed deaths, with 603 (61%) due to natural causes and 341 (34%) due to unnatural causes. Suicide was the most common unnatural death, contributing to 21% of all deaths. Cardiac death was the most common natural death, contributing to 19% of all deaths. The proportions of the leading causes of death contributing to all‐cause deaths in AN are presented in Table 4.

**TABLE 4:** The pooled proportion of the natural, unnatural and other leading causes of death in AN.

Cause of death	Natural	Unnatural	Suicide	Cardiac^a^	Pneumonia	Cancer	Hepatic^b^	Poison^c^
No. of studies combined	15	15	15	11	11	11	11	13
Total specific cause death	603	341	214	14	7	7	6	6
Total all‐cause death	994	994	994	77	77	77	77	85
Overall proportion by GLMM^d^	61%	32%	21%	19%	9.1%	9.1%	7.8%	7.1%
95% CI	56%–66%	24%–41%	14%–30%	8%–38%	4%–18%	4%–18%	3.5%–16%	3%–15%
General population^e^	78%	22%	9.2%	9%	1.8%	32%	3.8%	0.28%

*Note*: This table presents the pooled proportion of specific causes of death among all‐cause deaths.

^a^ Out of the 14 cardiac deaths, four were due to arrhythmia, four were due to heart failure, two were due to coronary insufficiency, and four were non‐specified.

^b^ Among six hepatic deaths, two were linked to alcohol use, and the others were not defined.

^c^ Among the six deaths by poison, four were due to alcohol intoxication.

^d^ The proportions from individual studies were pooled using the generalized linear mixed‐effect model (GLMM).

^e^ The general population's death percentage of each specific cause is estimated from the Global Burden of Disease 2019.

### 3.5 Outlier and Influence Analyses

Four study units were identified as outliers (Crisp and Collaborators 2006; Gueguen et al. 2012; Guinhut et al. 2021; Huas et al. 2011; Korndorfer et al. 2003). Recalculating the pooled SMR without the outliers reduced *I* ^2^ to 79.6%, increasing the SMR to 5.48, 95% CI (4.22–7.11). Influence analyses by the leave‐one‐out method were conducted. The Baujat plots (Supporting Information) showed that three studies, also previously identified as outliers, contributed the most to the heterogeneity (Crisp and Collaborators 2006; Guinhut et al. 2021; Korndorfer et al. 2003). No study is consistently identified as an influencer in the influence diagnostic plot (Supporting Information). The leave‐one‐out meta‐analysis revealed that omitting any single study had a minimal impact on the pooled effect size and heterogeneity (see Supporting Information).

### 3.6 Sensitivity Analysis

#### 3.6.1 Allowing Partial Sample Overlap for Pooling SMR

In this meta‐analysis, studies from the large patient registers were excluded to ensure study independence. Sensitivity analysis was done by allowing partial overlap and pooling all 28 study units (formed from 30 studies). The pooled SMR was 5.35, with a 95% CI (3.94–7.27), similar to the main result. The forest plot is presented as Supporting Information.

### 3.7 Publication Bias

**FIGURE 3:**
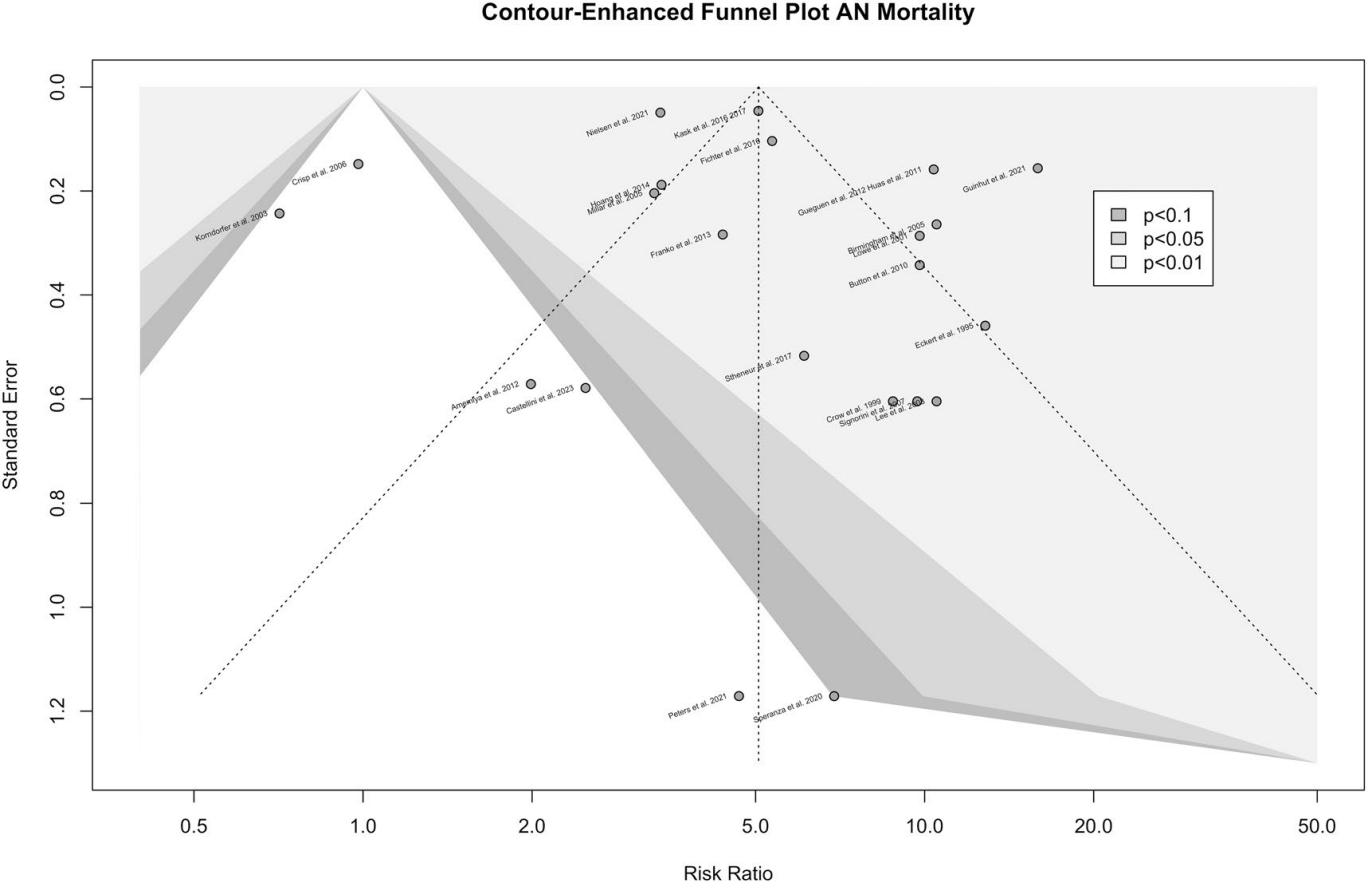
Funnel plot with contour enhancement for the 22 study unit pooled for all‐cause SMR. Visual inspection of the funnel plot showed that distributions were roughly symmetrical, indicating no publication bias. Two apparent outliers lie in the statistically insignificant area, likely due to between‐study heterogeneity. SMR, standardized mortality ratio. Egger's regression test showed the intercept was 0.62, 95% CI (−1.72–2.97), *p* = 0.61.

Visual inspection of the funnel plot showed that the studies were distributed roughly symmetrically (see Figure 3). Egger's regression test showed the resulting intercept was 0.62, with a 95% CI (−1.72–2.97) and *p* = 0.61, which did not indicate the presence of funnel plot asymmetry.

### 3.8 Sub‐Group Analysis

The difference in SMR between inpatients and studies with mixed settings was not significant from subgroup analysis (see Table 5). The differences in SMR found from studies using ICD‐10, DSM‐IV, and DSM‐III‐R were also found to be non‐significant (see Table 5). The subgroup analyses on study design and study quality were non‐significant (see Table 5).

**TABLE 5:** Subgroup analysis of treatment settings, diagnostic criteria, study design, study quality and sample gender.

	*k*	SMR	95% CI	$I^{2}$	*p*_subgroup_	Adjusted *p*_subgroup_
Treatment setting					0.126	1
Inpatient	10	6.68	4.25–10.48	89.1%		
Mixture of outpatient and inpatient	12	3.95	2.14–7.30	92.4%		
Diagnostic criteria					0.510	1
DSM‐III‐R	3	3.88	1.35–11.1	95.2%		
DSM‐IV	9	7.05	3.85–12.91	83.3%		
ICD‐10 (F50.0, F50.1)	2	3.69	0.89–15.32	0.0%		
Study design					0.07	0.91
Clinic‐based study	18	6.11	4.15–8.99	92.6%		
Population‐based study	4	2.60	0.67–10.14	96.6%		
Study quality					0.15	1
Good or fair	17	5.68	3.81–8.49	93.0%		
Poor	5	2.75	0.76–9.91	78.0%		
Study sample gender					0.730	1
Male	9	3.47	1.60–7.52	74.4%		
Female	9	3.86	1.82–8.20	97.1%		

Abbreviations: Adjusted *p*_subgroup_, *p*‐value of the subgroup analysis with Bonferroni corrections for multiple testing; *I* ^2^, residual heterogeneity; *p*_subgroup_, *p*‐value of the subgroup analysis; *k*, number of studies.

#### 3.8.1 Studies With Male‐Only Patients Compared With Female Patients

**FIGURE 4:**
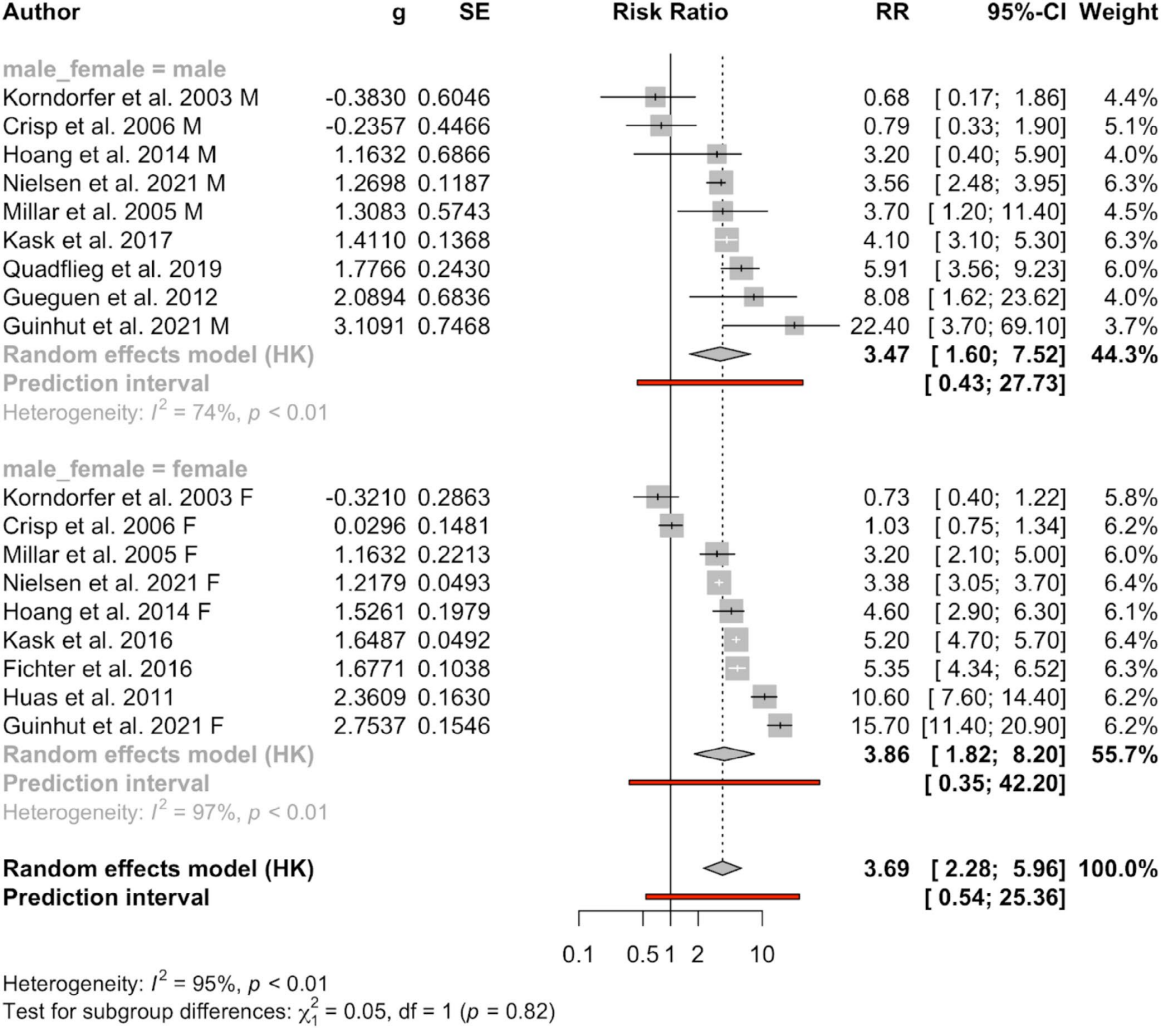
The forest plot of the subgroup analysis on gender difference in SMR. This is the forest plot of the subgroup analysis comparing studies that reported male‐specific SMRs with the female‐specific SMRs from the same cohort. Subgroup analysis of 9 study pairs showed that the pooled male SMR was 3.47, with a 95% CI (1.60–7.52), similar to the pooled female SMR of 3.86, with a 95% CI (1.82–8.20). The restricted maximum likelihood estimator was used for the between‐study effect size variance. AN, anorexia nervosa; HK, Hartung‐Knapp modification; SMR or RR, standardized mortality ratio.

Six study reports provided the male‐specific and female‐specific SMRs separately (Crisp and Collaborators 2006; Guinhut et al. 2021; Hoang et al. 2014; Korndorfer et al. 2003; Millar et al. 2005; Nielsen and Vilmar 2021). Another two male‐patient study samples were published separately from their female‐patient study samples, from Sweden (Kask et al. 2016, 2017) and France (Gueguen et al. 2012; Huas et al. 2011). Lastly, Quadflieg published a male‐only study recruited from a German hospital from 1985 to 2017 and a study with 98.4% females from the same hospital recruited from 1985 to 2005 (Fichter and Quadflieg 2016; Quadflieg et al. 2019). The subgroup analysis of 9 study pairs showed that the pooled male SMR was 3.47, 95% CI (1.60–7.52), similar to the pooled female SMR of 3.86, 95% CI (1.82–8.20). The result is presented in Table 5, and the forest plot in Figure 4. The funnel plot and Egger's test did not identify publication bias (see Supporting Information).

### 3.9 Meta‐Regression Analysis

**FIGURE 5:**
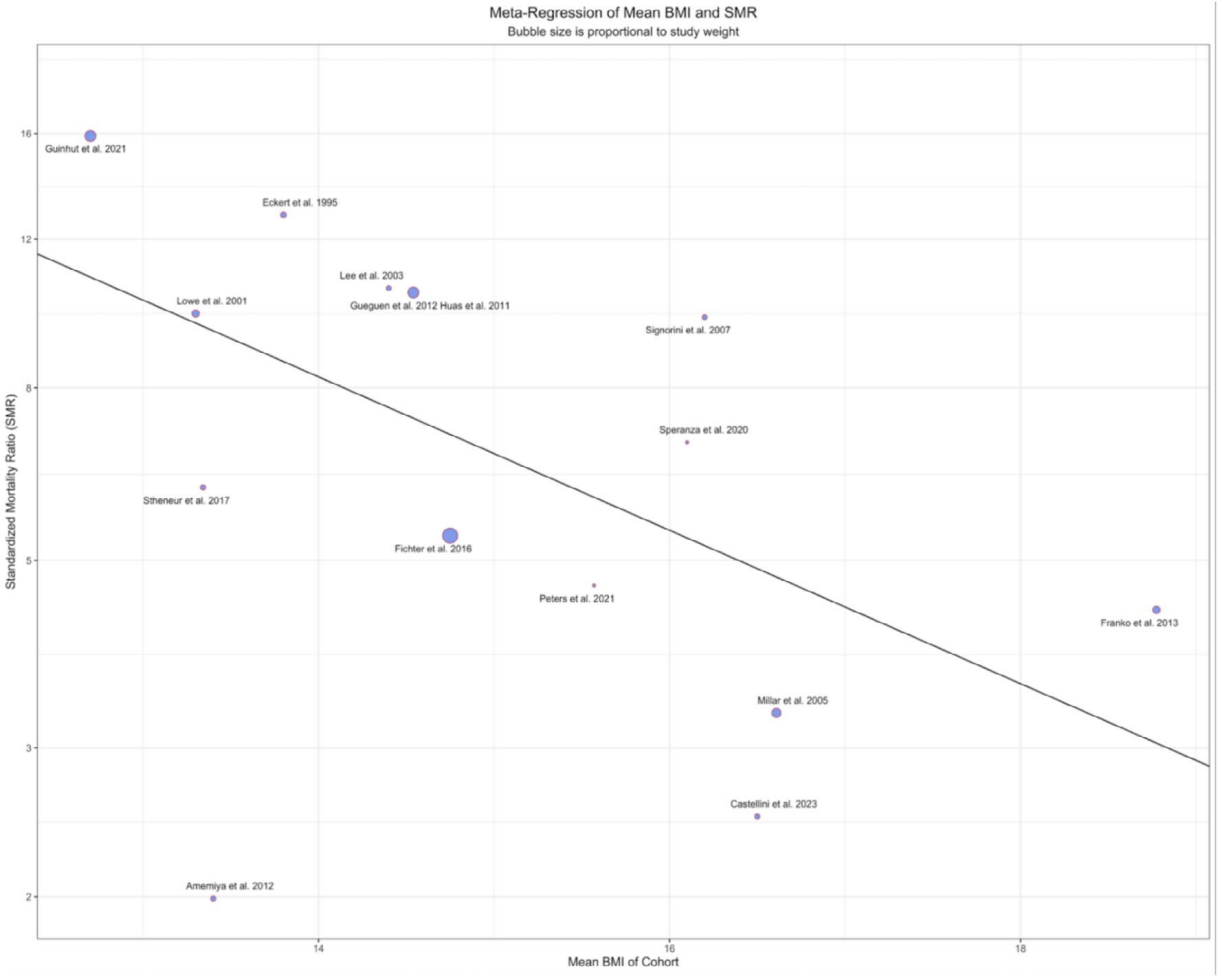
Meta‐regression analysis for log SMR vs. study sample mean BMI. The meta‐regression analysis of log SMR versus the cohort's mean BMI at study entry showed a significant association (*p* = 0.018).

Bivariate meta‐regressions were done for nine predictors (see Table 6). The sample mean BMI as a predictor for SMR was statistically significant before correcting for multiple testing (*β* = −0.209, 95% CI (−0.376, −0.042), *p* = 0.018, adjusted *p* = 0.252; see Table 6, Figure 5). Furthermore, the sample mid‐recruitment year as a predictor for SMR was statistically significant before correcting for multiple testing (*β* = 0.038, 95% CI (0.009, −0.067), *p* = 0.014, adjusted *p* = 0.196; see Table 6). Lastly, the study duration as a predictor for SMR was statistically significant before correcting for multiple testing (*β* = −0.0290, 95% CI (−0.052, −0.006), *p* = 0.018, adjusted *p* = 0.252; see Table 6). Other predictors, that is, mean age, mean duration of follow‐up, sample size, ascertainment rate, percentage of patients with psychiatric comorbidities, and percentage of patients with binge‐purging subtype, were statistically non‐significant (see Table 6).

**TABLE 6:** Meta‐regression analysis of SMR predictors in studies on anorexia nervosa.

Predictor	*k*	$I^{2}$	*p*_residual_	$R^{2}$	β (95% CI)	*p*_moderators_	Adjusted *p*_moderators_
Mean age	18	95.01%	< 0.0001	13.65%	0.067 (−0.060–0.194)	0.278	1
Mean BMI	14	54.98%	0.0011	64.79%	−0.209 (−0.376–0.042)	0.018*	0.252
Mid recruitment year	21	94.59%	< 0.0001	37.23%	0.038 (0.009–0.067)	0.014*	0.196
Mean duration of follow‐up	15	91.26%	< 0.0001	22.52%	−0.051 (−0.115–0.014)	0.116	1
Study duration	21	94.6%	< 0.0001	27.76%	−0.0290 (−0.052–0.006)	0.018*	0.252
Sample size	22	95.77%	< 0.0001	2.41%	−0.000 (−0.000–0.000)	0.485	1
Ascertainment rate	20	96.46%	< 0.0001	5.35%	4.339 (−3.183–11.861)	0.241	1
% of patients with psychiatric comorbidities	6	86.92%	< 0.0001	3.82%	−0.008 (−0.048–0.033)	0.627	1
% of patients with binge‐purging subtype	12	64.87%	0.0004	25.61%	−0.012 (−0.036–0.011)	0.274	1

*Note*: *p < 0.05.

Abbreviations: 95% CI, The estimated change in the outcome (ln SMR) for one‐unit increase in the moderator; Adjusted *p*_moderators_: *p*‐value of the test of moderators with Bonferroni corrections for multiple testing; *I* ^2^, residual heterogeneity; *R* ^2^, the amount of heterogeneity accounted for; *p*_moderators_: *p*‐value of the test of moderators; *p*_residual_: *p*‐value of the test for residual heterogeneity.

In view of the strong negative correlation between the mid recruitment year and study duration (*r* = −0.69, *p* = 0.001), both moderators were included in a multivariable meta‐regression model to assess their independent effects. The overall multivariable model was statistically significant (*p* = 0.030; see Table 7). Within this model, however, neither the midpoint of recruitment year (*β* = 0.024, 95% CI (−0.015, 0.063), *p* = 0.209; see Table 7) nor total study duration (*β* = −0.016, 95% CI (−0.048, 0.016), *p* = 0.296; see Table 7) was a statistically significant independent predictor of SMR.

**TABLE 7:** Multivariate meta‐regression analysis of SMR predictors in studies on anorexia nervosa.

Predictor	*k*	$I^{2}$	*p*_residual_	$R^{2}$	β (95% CI)	pvalue
Overall model	21	93.51%	< 0.0001	39.33%		0.030*
Mid recruitment year					0.024 (−0.015–0.063)	0.209
Study duration					−0.016 (−0.048–0.016)	0.296

*Note*: Multivariate meta‐regression analysis was used to investigate the independent effects of mid‐recruitment year and study duration on the SMR. The overall model was statistically significant, but the coefficients of each predictor were statistically non‐significant. *p < 0.05.

Abbreviations: 95% CI, the estimated change in the outcome (ln SMR) for a one‐unit increase in the moderator; *I* ^2^, residual heterogeneity; *k*, number of studies; *p*_residual_, *p*‐value of the test for residual heterogeneity; *R* ^2^, the amount of heterogeneity accounted for.

### 3.10 Qualitative Synthesis of Psychiatric Comorbidities and Mortality

Among the included studies, only four study units reported the percentage of any psychiatric comorbidities, and therefore their effect on mortality could not be examined quantitatively (Guinhut et al. 2021; Kask et al. 2016, 2017; Peters et al. 2021; Rosling et al. 2011). The effect of psychiatric comorbidities on mortality for patients with AN was qualitatively synthesized through examining the included studies' results. Among the included studies, two Swedish registry studies on AN patients with an age limit of 10–40 years old highlighted the increase in mortality in AN patients with psychiatric comorbidities (Kask et al. 2016, 2017). The Swedish female AN population study showed that the SMR for patients with AN only was 3.2, 95% CI (2.7–3.9), while the SMR for patients with other psychiatric comorbidities was 8.2, 95% CI (7.3–9.2). The highest SMR was from alcohol use disorder 17, 95% CI (13.9–20.5) and other substance use disorder 18.3, 95% CI (14.7–22.6) (Kask et al. 2016). The result from the Swedish male AN population showed a similar effect, with the SMR for patients with AN only being 1.6, 95% CI (0.9–2.7), while the SMR for patients with any other psychiatric comorbidities was 9.1, 95% CI (6.6–12.2) (Kask et al. 2017). The highest SMR was also from alcohol use disorder 18.9, 95% CI (11.4–29.5) and other substance use disorder 28.4, 95% CI (13.6–52.2). Another registry based on Southeast London's data, with no age limit imposed, showed substance use disorder and personality disorder (PD) predicted increased SMR (Himmerich et al. 2019). However, these two factors were insignificant after controlling for age, sex, ethnicity, borough, marital status, and deprivation score. Two studies on clinical samples supported alcohol misuse as a predictor for increased mortality (Button et al. 2010; Franko et al. 2013). The results from the Swedish registers, described in the above paragraph, showed that the SMR for female patients with AN and PD was 10.7, 95% CI (8.8–12.9), and that for male patients was 9.6, 95% CI (5.3–16.2) (Kask et al. 2016, 2017).

## 4 Discussion

This systematic review and meta‐analysis is currently the most comprehensive study on the mortality of AN, including 30 studies up to 2025 and an extensive moderator analysis. The primary finding is that AN is associated with a five‐fold increase in mortality risk compared to the general population (pooled all‐cause SMR = 5.06). However, there is substantial between‐study heterogeneity resulting in a wide 95% prediction interval, suggesting that outcomes for specific cohorts can differ significantly from the average. Crucially, as this interval includes the null value of 1, it indicates that some cohorts of individuals with AN may not experience elevated mortality. Suicide was the leading cause of death (21%), followed by cardiac complications (19%) and pneumonia (9.1%). Our analysis preliminarily suggested that mortality was higher in studies with lower mean BMI at study entry. This systematic review was the first to examine gender differences in mortality; however, the SMR of male‐specific samples was shown to be similar to that of female‐specific samples.

Our study found a pooled SMR of 5.06, which was consistent with previous meta‐analyses published in 2011 and 2025, reporting SMRs of 5.86 and 5.21, respectively (Arcelus et al. 2011; Krug et al. 2025). Among other mental disorders, AN's mortality is below that of opioid use disorder, similar to other substance use disorders, and higher than schizophrenia or depression (Chesney et al. 2014). This underscores that AN is a serious mental condition.

Our analysis revealed that suicide accounted for 21% of all deaths in AN, making it the leading cause of mortality. This proportion is consistent with a prior estimate in AN (Arcelus et al. 2011). Compared to other mental disorders, the percentage of deaths due to suicide in AN is similar to that of personality disorder (28%), higher than that of substance use disorder (10%) and bipolar disorder (7.2%) (Crump et al. 2013; Hjemsæter et al. 2019; Høye et al. 2021). The link between eating disorders and suicide has been attributed to comorbid mood disorder (Bodell et al. 2013), shared genetic factors (Thornton et al. 2016), and increased exposure and reduced fear of painful and destructive behaviors (Selby et al. 2010; Zeppegno et al. 2021). These findings underscore the importance of screening for suicidality and integrating evidence‐based suicide prevention strategies, such as cognitive behavioral therapy for suicide prevention or safety planning, into the standard AN treatment (Nuij et al. 2021; Wu et al. 2022).

Cardiac complications were the second most common cause of death, contributing to 19% of fatalities. AN is associated with several cardiac abnormalities, for example, bradycardia, QT interval prolongation, reduced cardiac mass, mitral valve prolapse, and mild pericardial effusion (Giovinazzo et al. 2019). AN‐associated behaviors, that is, purging and intensive exercise, can cause QT interval prolongation. These AN‐associated cardiac changes and behaviors may contribute to cardiac death (Jauregui‐Garrido and Jauregui‐Lobera 2012). To address these risks, clinicians should be vigilant for electrolyte disturbance and signs of cardiac complications. In addition, psychotropics that prolong the QT interval should be used judiciously. Furthermore, early treatment targets for individuals with AN should prioritize reducing behaviors that increase the risk of arrhythmia, such as purging or excessive exercise.

Pneumonia accounted for 9.1% of all deaths in this meta‐analysis, higher than the 1.8% seen in the general population. The increased risk of pneumonia in AN is not attributed to malnutrition‐related immune suppression (Słotwińska and Słotwiński 2017) but rather to aspiration from oropharyngeal dysphagia or susceptibility to non‐tuberculosis mycobacteria pulmonary infection (Holmes et al. 2016; Nitsch et al. 2023). Therefore, timely detection of oropharyngeal dysphagia and signs of chest infection may reduce these fatalities.

A low BMI has been a cardinal feature of AN and is used in the DSM‐5 for severity classification (American Psychiatric Association 2022). This feature is correlated with pathological changes, that is, bradyarrhythmia, lower left ventricular mass, delayed gastric emptying (Bluemel et al. 2017; Farasat et al. 2020; Smythe et al. 2021), as well as suicidality (Arnold et al. 2023; Favaro and Santonastaso 1997). However, the precise relationship between BMI and mortality has been a subject of debate. While several large cohort studies found that a low BMI at study entry predicts a higher SMR (Button et al. 2010; Fichter and Quadflieg 2016; Franko et al. 2013; Gueguen et al. 2012), two major meta‐analyses did not find a significant association, concluding that BMI may not influence mortality risk (Arcelus et al. 2011; Krug et al. 2025). These negative findings, along with other studies demonstrating that lower BMI is not associated with more severe eating psychopathology (Machado et al. 2017; Smith et al. 2017; Zayas et al. 2018), question the utility of BMI as a primary severity indicator for AN (Dang et al. 2022, 2023). Alternative severity indicators such as weight suppression and drive for thinness have been proposed for AN (Krug et al. 2021; Lowe et al. 2018). Our meta‐regression helps to clarify these conflicting findings. We identified a trend suggesting that studies with a lower mean entry BMI reported higher SMRs. However, this association did not reach the stringent threshold for statistical significance after correcting for multiple testing and must therefore be considered preliminary. Nonetheless, this result shows that BMI as a severity indicator of AN cannot be dismissed.

The study‐level association between lower BMI and higher SMR was not detected in previous meta‐analyses for two reasons. First, the more recent analysis by Krug et al. (2025) was likely underpowered to detect this effect, as BMI data were extracted from only five of their included AN studies. Second, compared to the landmark meta‐analysis by Arcelus et al. (2011), our meta‐regression included more recent studies that capture more extreme BMI values at both ends of the weight spectrum (Amemiya et al. 2012; Franko et al. 2013; Guinhut et al. 2021; Stheneur et al. 2017) and used a recalculated baseline of 13.8 kg/m^2^ for the Eckert et al. (1995) cohort, based on its primary data (Halmi et al. 1979). This cohort was previously analyzed using a BMI of 17.4 kg/m^2^ in Arcelus et al. (2011), likely due to an extraction error. These refinements provide greater statistical power, suggesting the prior null finding may be a product of these data limitations rather than a true lack of association. Given the results of this updated analysis, it remains clinically prudent to consider a low BMI as a key indicator of risk, alongside other physical, behavioral, and psychological factors. Nonetheless, our result could also be confounded by other proposed severity indicators, such as weight suppression, which were not commonly measured in previous longitudinal studies. In conclusion, our study reinforces the importance of early detection and supports clinical guidelines that identify a very low BMI as a critical factor for prioritizing patients for specialist eating disorder services (Hilbert et al. 2017). Future longitudinal studies should investigate the independent predictive value of BMI and other proposed severity indicators for mortality risk in AN (Dang et al. 2022).

In post hoc exploratory analyses, we investigated temporal changes in the SMR of patients with AN, given advances in psychotherapeutic treatments. Using the mid‐point of study recruitment as a proxy for the treatment era, a univariable meta‐regression yielded the counterintuitive finding that more recent studies were associated with higher SMRs. However, this relationship appears to be a statistical artifact driven by confounding from study duration. We found that more recent recruitment years were strongly correlated with shorter study durations, and shorter durations were, in turn, significantly associated with higher SMRs. This latter finding is well established, as the mortality risk in AN is highest in the initial period following diagnosis (Franko et al. 2013; Huas et al. 2011; Papadopoulos et al. 2009; Rosling et al. 2011; Suokas et al. 2013). When both recruitment year and study duration were included in a multivariable model, the effect of recruitment year was no longer significant. Therefore, after accounting for methodological differences in study follow‐up length, our analysis provides no evidence of a genuine temporal trend in mortality for patients with AN.

The mortality for males with AN has been understudied, with the last systematic review showing inconclusive results (Strobel et al. 2018). Our meta‐analysis found that male AN had a similar SMR compared to that of the female counterpart. Given that most research on AN focuses on females, there is an urgent need to understand and address the clinical needs of male AN.

Most patients with AN present with additional psychiatric diagnoses, with substance use, alcohol use, and mood disorders being the most common comorbidities (Qeadan et al. 2023; Root et al. 2010; Udo and Grilo 2019). Qualitative synthesis from this study showed that comorbid alcohol and substance use, and personality disorders increased mortality in AN. This is further substantiated by our own data, which showed that hepatic and poison deaths contributed to 7.8% and 7.1% of the total deaths, and both causes were mainly related to alcohol use (See Table 4). The heightened risk of fatal alcohol poisoning in this population may be explained by a specific physiological vulnerability. Research indicates that even in weight‐restored patients with AN, fat‐free mass restoration can be inadequate, resulting in lower total body water volume compared to healthy individuals (dos Reis et al. 2022; Hübel et al. 2019). This lower water volume causes any given dose of alcohol to produce a higher peak blood alcohol concentration, thereby increasing the risk for a fatal overdose (Kalant 2000; Watson et al. 1981). These findings highlight the importance of clinicians screening for and managing comorbidities in patients with AN. Despite the high prevalence of psychiatric comorbidities and increased risk, there is a notable lack of evidence‐based interventions specifically for this patient group. This points to an urgent need to develop and test integrated, transdiagnostic treatments to address this vulnerable group. Approaches that target underlying shared mechanisms, such as dialectical behavior therapy (DBT), which addresses emotion dysregulation, are promising candidates that warrant further investigation (Claudat et al. 2020; Linardon et al. 2019).

This meta‐analysis has several strengths worth highlighting. Firstly, this was the most comprehensive systematic review and meta‐analysis of mortality for people with AN to date. Secondly, a comprehensive literature search was done using multiple databases. Thirdly, all studies were meticulously scrutinized to ensure study independence and data accuracy. Lastly, extensive sociodemographic, clinical, and methodological moderators were extracted from the included studies, allowing for thorough analysis of the between‐study heterogeneity in AN mortality.

This study has several limitations that must be acknowledged. Firstly, our meta‐analysis was restricted to studies that reported an SMR. This necessarily led to the exclusion of many reports, particularly those with zero deaths, which often do not calculate an SMR. While many of these excluded studies had methodological limitations of their own (e.g., short follow‐up periods, small sample sizes), their exclusion may have biased our pooled SMR upwards. Therefore, our estimated mortality rate should be interpreted as being most representative of cohorts with at least some mortality events, rather than the entire spectrum of AN outcomes. Secondly, the heterogeneity of the included studies was substantial. Although we explored several moderators, most of the variance remained unexplained. The primary impact of this is on the interpretation of our pooled SMR. The wide prediction interval, which includes the null effect, underscores that while the average mortality risk is high across studies, the risk for any single future population is highly uncertain. This high heterogeneity indicated that AN mortality is likely influenced by a complex interplay of factors not captured in this analysis. Thirdly, our subgroup and meta‐regression analyses involved multiple statistical tests. We have presented both uncorrected and corrected *p*‐values to ensure transparency regarding our exploratory findings. The fact that our key meta‐regression of BMI did not survive correction for multiple testing means it must be considered preliminary. Furthermore, most studies lack reporting of race, ethnicity, and socioeconomic status. Lastly, the generalizability of our findings is constrained. The exclusion of non‐English studies, the underrepresentation of male and non‐Western populations, and the fact that most studies are from AN specialist units mean our results are most applicable to female patients in Western, specialist treatment settings. The mortality risk and causes of death may differ significantly in primary care settings, in other cultures, or among males, whose mortality outcomes remain poorly understood.

Despite its limitations, this meta‐analysis provides critical insights for both clinicians and researchers. Our findings confirm that AN has a high mortality risk, with suicide being the leading cause of death, implying that suicide risk assessment and prevention must be a central component of AN care. Furthermore, the identification of cardiac and pneumonia as major causes of death underscores the need for proactive medical monitoring, including for cardiac complications, dysphagia that can lead to aspiration pneumonia, and signs of chest infection. Our preliminary finding linking lower BMI to higher SMR reinforces the clinical prudence of using BMI as a key indicator of medical risk and supports guidelines that prioritize individuals with very low BMI for intensive treatment and specialist services.

This meta‐analysis also revealed several critical gaps in the research literature. Firstly, our findings highlight the crucial need for more population‐based studies. Our preliminary subgroup analysis found a trend toward lower SMRs in population‐based cohorts compared to clinic‐based samples. While this analysis was underpowered, it suggests that mortality risk may be overestimated in a literature dominated by specialist clinic samples. Therefore, more population‐based studies would clarify the difference in risk between patients managed in primary care versus specialist settings. Secondly, more large‐scale studies on mortality outcomes for male AN patients are required to understand their specific risk factors and prognosis. Thirdly, further studies on the AN populations with psychiatric comorbidities are required to understand their synergistic effect on mortality. Furthermore, after establishing suicide as the leading cause of death, further work should focus on co‐creating specific suicide prevention strategies tailored to the unique psychological profile of individuals with AN (Krysinska et al. 2023; Papastavrou Brooks et al. 2023). Lastly, longitudinal studies are needed to delineate the long‐term effects of AN on cardiac health and to identify strategies to mitigate the risk of cardiac death beyond weight restoration.

## Author Contributions

**Eric Tsz‐Him Lai:** conceptualization, investigation, writing – original draft, methodology, visualization, writing – review and editing, formal analysis, project administration, data curation. **Benjamin Lai:** investigation, data curation. **Corine Sau‐Man Wong:** methodology, writing – review and editing, supervision. **Lai‐Yi Wong:** conceptualization, writing – review and editing. **Kin‐Shing Cheng:** conceptualization, writing – review and editing. **Pak‐Wing Calvin Cheng:** writing – review and editing. **Lo Heidi Ka‐Ying:** writing – review and editing. **Gary Tse:** writing – review and editing. **Wai‐Chi Chan:** writing – review and editing. **Wing‐Chung Chang:** writing – review and editing. **Ka‐Fai Chung:** supervision, writing – review and editing.

## Funding

The authors have nothing to report.

## Ethics Statement

Ethical approval was not required for this study as it is a systematic review and meta‐analysis of previously published data.

## Consent

The authors have nothing to report.

## Conflicts of Interest

The authors declare no conflicts of interest.

## Supporting information


**Data S1:** PRISMA_checklist.

**Data S2:** eat70002‐sup‐0002‐supinfo.docx.

## Data Availability

The data that support the findings of this study are available from the corresponding author upon reasonable request.
