# Supplementary material for: Systematic Review and Meta‐Analysis of Mortality in Patients With Anorexia Nervosa
Source: Int J Eat Disord. 2025 Nov 23;59(3):424–49. doi: 10.1111/eat.70002 (PMC12979973; doi:10.1111/eat.70002)
Supplement: Supplementary file 2 — Data S2: eat70002‐sup‐0002‐supinfo.docx. [file EAT-59-424-s002.docx]

SUPPLEMENTARY MATERIALS

1. Search Strategies
2. Extracted Data
3. Quality assessment- Newcastle-Ottawa Scale Adapted
4. Required Sample Size for Cohort Study on AN Mortality
5. Assessing the Certainty of Evidence
6. Multiple Reports of Selected Studies and Overlapping Studies
7. Non-English Studies excluded
8. Outlier and Influence Analyses Figures
9. Sensitivity Analysis Figures
10. Figures for the Analysis of Studies with Male-only Patients Compared with Female Patients

##

## 1. Search Strategies

### Initial Search

The first round of the search was between August 2022 and December 2022.

***Ovid MEDLINE(R) <1946 to August Week 3 2022>***

*1 anorexia nervosa.mp. or exp Anorexia Nervosa/ 16670*

*2 exp Mortality/ or exp Survival analysis/ or exp Survival rate/ or exp Cause of death/ or Mortality.mp. or Fatality.mp. or Survival.mp. or Death.mp. 2679151*

*3 (Mortality or Fatality or Survival or Death).mp. 2595580*

*4 1 and (2 or 3) 929*

*5 eating disorder*.mp. or exp "Feeding and Eating Disorders"/ 39530*

*6 (1 or 5) and (2 or 3) 1615*

***Embase <1974 to 2022 August 26>***

*1 anorexia nervosa.mp. or exp Anorexia Nervosa/ 24266*

*2 exp Mortality/ or exp Survival analysis/ or exp Survival rate/ or exp Cause of death/ or Mortality.mp. or Fatality.mp. or Survival.mp. or Death.mp. 4320470*

*3 (Mortality or Fatality or Survival or Death).mp. 4320465*

*4 1 and (2 or 3) 1808*

*5 eating disorder*.mp. or exp eating disorder/ 63583*

*6 (1 or 5) and (2 or 3) 3505*

***ProQ PsycINFO, searched on 20/12/22***

*(MJMAINSUBJECT.EXACT.EXPLODE("Eating Disorders") OR "Eating disorders" OR "Eating disorder" OR MJMAINSUBJECT.EXACT.EXPLODE("Anorexia Nervosa") OR "Anorexia Nervosa") AND (MJMAINSUBJECT.EXACT.EXPLODE("Mortality Risk") OR MJMAINSUBJECT.EXACT.EXPLODE("Mortality Rate") OR mortality OR fatality OR survival OR death)*

*Methodology*

*Clinical Case Study, Clinical Trial, Empirical Study, Followup Study, Longitudinal Study, Prospective Study, Qualitative Study, Quantitative Study, Retrospective Study,*

*Treatment Outcome*

*Population, Human*

This search returned 791 articles.

***ProQuest Dissertations & Theses A&I (PQDT), searched on 21/12/22***

noft(("anorexia nervous" OR "eating disorder" OR "eating disorders") AND (Mortality OR Fatality OR Survival OR Death))

The dissertations were searched everywhere except full text because of the low yield of search terms from the full text, which invariably provides an extensive review of the topic. This search returned 204 articles.

### First update Search

The first updated search was conducted after 1 April 2023. It was decided to include Web of Science and Google Scholar to increase the comprehensiveness of the search. Furthermore, I have rerun the search of PsycINFO from inception to drop the filters on methodology and population for the search strategies to be more homogenous across databases.

The search strategies were as follows:

***Ovid MEDLINE(R) <1946 to March Week 4 2023>***

*1 anorexia nervosa.mp. or exp Anorexia Nervosa/ 16995*

*2 exp Mortality/ or exp Survival analysis/ or exp Survival rate/ or exp Cause of death/ or Mortality.mp. or Fatality.mp. or Survival.mp. or Death.mp. 2754616*

*3 (Mortality or Fatality or Survival or Death).mp. 2670798*

*4 1 and (2 or 3) 960*

*5 eating disorder*.mp. or exp "Feeding and Eating Disorders"/ 40661*

*6 (1 or 5) and (2 or 3) 1663*

*7 limit 6 to ed=20220801-20230401 50*

MEDLINE had 50 further articles.

***Embase <1974 to 2023 April 03>***

*1 anorexia nervosa.mp. or exp Anorexia Nervosa/ 25374*

*2 exp Mortality/ or exp Survival analysis/ or exp Survival rate/ or exp Cause of death/ or Mortality.mp. or Fatality.mp. or Survival.mp. or Death.mp. 4597805*

*3 (Mortality or Fatality or Survival or Death).mp. 4597793*

*4 1 and (2 or 3) 1923*

*5 eating disorder*.mp. or exp eating disorder/ 67694*

*6 (1 or 5) and (2 or 3) 3784*

*7 limit 6 to dc=20220801-20230401 288*

Embase had 288 further articles.

***ProQuest PsycINFO search before 1 April 2023***

*((MJMAINSUBJECT.EXACT.EXPLODE("Eating Disorders") OR "Eating disorders" OR "Eating disorder" OR MJMAINSUBJECT.EXACT.EXPLODE("Anorexia Nervosa") OR "Anorexia Nervosa") AND (MJMAINSUBJECT.EXACT.EXPLODE("Mortality Risk") OR MJMAINSUBJECT.EXACT.EXPLODE("Mortality Rate") OR mortality OR fatality OR survival OR death))*

PsycINFO gives 1901 articles. Compared with the previously included study from PsycINFO using Zotero, there were 1110 extra articles. Removing five detected duplications gives 1105 additional articles.

***ProQuest Dissertations & Theses A&I***

*noft("anorexia nervosa" OR "eating disorder" OR "eating disorders") AND noft(Mortality OR Fatality OR Survival OR Death)*

This search returned three extra studies

***Web of Science core collection***

*(ALL=(anorexia nervosa or "eating disorder*")) AND ALL=(Mortality or Fatality or Survival or Death)*

*From 1900 to 2023-04-01*

This search returned 2538 results. Among those, there were 11 duplicated articles detected.

***Google Scholar***

("anorexia nervosa" OR "eating disorder" OR "eating disorders") AND (Mortality OR Fatality OR Survival OR Death)

The first 200 results were exported as suggested (Bramer et al., 2017).

### Second Update Search

A second update search was done in May 2025. The search strategies were as follows:

**MEDLINE**

Ovid MEDLINE(R) <1946 to May Week 2 2025>

1 anorexia nervosa.mp. or exp Anorexia Nervosa/ 18154

2 exp Mortality/ or exp Survival analysis/ or exp Survival rate/ or exp Cause of death/ or Mortality.mp. or Fatality.mp. or Survival.mp. or Death.mp. 3032119

3 (Mortality or Fatality or Survival or Death).mp. 2945781

4 1 and (2 or 3) 1047

5 eating disorder*.mp. or exp "Feeding and Eating Disorders"/ 44576

6 (1 or 5) and (2 or 3) 1840

7 limit 6 to ed=20230401-20250501 171

To: Update_ANmortality_MEDLINE_20250501.ris

**EMBASE**

Embase Classic+Embase <1947 to 2025 May 20>

1 anorexia nervosa.mp. or exp Anorexia Nervosa/ 29331

2 exp Mortality/ or exp Survival analysis/ or exp Survival rate/ or exp Cause of death/ or Mortality.mp. or Fatality.mp. or Survival.mp. or Death.mp. 5391386

3 (Mortality or Fatality or Survival or Death).mp. 5391344

4 1 and (2 or 3) 2233

5 eating disorder*.mp. or exp eating disorder/ 80686

6 (1 or 5) and (2 or 3) 4613

7 limit 6 to dc=20230401-20250501 817

**Proquest PsycInfo**

((MJMAINSUBJECT.EXACT.EXPLODE("Eating Disorders") OR "Eating disorders" OR "Eating disorder" OR MJMAINSUBJECT.EXACT.EXPLODE("Anorexia Nervosa") OR "Anorexia Nervosa") AND (MJMAINSUBJECT.EXACT.EXPLODE("Mortality Risk") OR MJMAINSUBJECT.EXACT.EXPLODE("Mortality Rate") OR mortality OR fatality OR survival OR death)) AND pd(20230401-20250501)

Returning 165 articles.

***ProQuest Dissertations & Theses A&I***

noft("anorexia nervosa" OR "eating disorder" OR "eating disorders") AND noft(Mortality OR Fatality OR Survival OR Death)

Filter 20230401 to 20250501

Returning 20 articles

***Web of Science core collection***

(ALL=(anorexia nervosa or "eating disorder*")) AND ALL=(Mortality or Fatality or Survival or Death)

Timespan: 2023-04-01 to 2025-05-01 (Publication Date)

**507** results from Web of Science Core Collection

***Google Scholar***

("anorexia nervosa" OR "eating disorder" OR "eating disorders") AND (Mortality OR Fatality OR Survival OR Death)

The first 200 results were exported as suggested (Bramer et al., 2017).

## 2. Extracted data

#

Multiple data were extracted into a predefined spreadsheet to explore the differences between studies. These include year of publication, location of study, source of data, number of AN at entry, number of AN with vital status ascertained, study period, mean/median duration of follow-up, diagnostic criteria used, percentage of patients with psychiatric comorbidities, method of assessment of AN diagnosis, mean/ median age of the population studied at entry, mean years of eating disorder at study entry, percentage of male, percentage of binge purging subtype, mean BMI at study entry, treatment setting at recruitment, number of death, expected death, SMR, 95% confidence interval, number of natural death, number of unnatural death, number of unknown cause of death, number of death due to suicides, number of death due to poison, number of death due to accident, number of death due to cardiac cause, number of death due to pneumonia, number of death due to cancer, number of death due to liver disease, number of death due to upper gastrointestinal disease, number of death due to diabetes mellitus.

Complete data extracted will be made available upon request.

## 3. Quality assessment- Newcastle-Ottawa Scale Adapted

**Selection**

1. Representativeness of the Exposed Cohort
   1. Truly representative of the average patient with anorexia nervosa in the community* (community sample, registry data of both inpatient and outpatient)
   2. Somewhat representative of the average patient with anorexia nervosa in the community* (eating disorder service to both inpatient and outpatient)
   3. Selected group of users eg HIV+, pregnant, elderly, significant physical disabilities (Patients discharged from inpatient, Patients attended emergency department, follow-ed up of RCT)
   4. No description of the derivation of the cohort
2. Selection of the Non-Exposed Cohort
   1. Drawn from the same community as the exposed cohort*
   2. Drawn from a different source
   3. No description of the derivation of the non-exposed cohort
3. Ascertainment of Exposure
   1. Secure record (eg, medical records)*
   2. Structured interview *
   3. Written self-report, or just based on diagnostic coding without reviewing case note
   4. No description
4. Demonstration that Outcome of Interest Was Not Present at the start of Study
   1. Yes*
   2. No

**Comparability**

1. Comparability of Cohorts on the Basis of the Design or Analysis
   1. Study controls for age and gender*
   2. Study controls for length of follow-up*
   3. Inadequate degree of control

**Outcome**

1) Assessment of outcome

a) Independent blind assessment*

b) Record linkage*

c) Self report

d) No description

2) Was follow-up long enough for outcomes to occur (which is for expected death to be at least 1, see supplementary material section 4).

a) Yes*

b) No

3) Adequacy of follow-up of cohorts

a) Complete follow-up: all subjects accounted for *

b) Subjects lost to follow-up unlikely to introduce bias- number lost less than or equal to 10% or description of those lost suggested no different from those followed. *

c) Follow up rate less than 90% and no description of those lost

d) No statement

Thresholds for converting the Newcastle-Ottawa scales to The Agency for Healthcare Research and Quality (AHRQ) standards (good, fair, and poor quality studies) (Shamsrizi et al., 2020):

- “Good quality: 3 or 4 stars in selection domain AND 1 or 2 stars in comparability domain AND 2 or 3 stars in outcome/exposure domain”
- “Fair quality: 2 stars in selection domain AND 1 or 2 stars in comparability domain AND 2 or 3 stars in outcome/exposure domain”
- “Poor quality: 0 or 1 star in selection domain OR 0 stars in comparability domain OR 0 or 1 stars in outcome/exposure domain”

##

## 4. Required Sample Size for Cohort Study on AN Mortality

The required sample size, in the unit of person-year, to detect a significant SMR in AN mortality cohort study had not been described in the literature. Therefore, it was derived in this section to guide this study's quality assessment. The number of deaths in a cohort of patients with anorexia nervosa followed a Poisson distribution. As the observed death was compared with the expected death, derived from whole population mortality data, this was a one-sample inference problem.

We would first start with deriving the one-sample version of Lehr’s equation, which estimates the sample size required for testing for the mean of a normal distribution, before attending to the sample size required for testing a Poisson distribution.

**Sample Size Estimation when Testing for the Mean of a Normal Distribution**

**(One sample, two-sided test)**

Suppose we wish to test $H_{0}: \mu=\mu_{0} vs. H_{1}:\mu=\mu_{1}$, with the data normally distributed with mean 𝜇 and variance $\sigma^{2}$, the sample size needed (Rosner, 2016)

$n = \frac{\sigma^{2}(z_{1-\beta}+z_{1-\alpha/2})^{2}}{(\mu_{0}-\mu_{1})^{2}}$

Using the standard value of $\alpha=0.05, 1-\beta=80\%$, we have $z_{1-\alpha/2}=1.96$ and $z_{1-\beta}=0.84$

We have $n=\frac{8}{\Delta^{2}}$, where $\Delta=\frac{\mu_{0}-\mu_{1}}{\sigma}$, which is the standardised difference. This formula is the one-sample version of Lehr’s equation.

With this equation, we can apply it to the Poisson distribution.

**Sample Size Estimation when Testing for the Mean of a Poisson Distribution**

**(One sample, two-sided test)**

A Poisson distribution can be square-root transformed and approximated by a normal distribution^^[[1]](#footnote-0)^^.


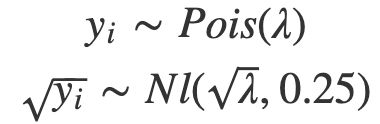


Applying the one sample version of Lehr’s equation derived above, we have

$n=\frac{8}{\left[ \left( \sqrt{\lambda_{1}}- \sqrt{\lambda_{2}} \right)/\sqrt{0.25} \right]^{2}}=\frac{2}{({\sqrt{\lambda_{1}}- \sqrt{\lambda_{2}})}^{2}{}}$

Which in our context, $\lambda_{1} and \lambda_{2}$ are the number of deaths per person-year in the anorexia nervosa population and general population respectively.

We can see that the incidence rate ratio, $IRR = \frac{\lambda_{1}}{\lambda_{2}}$.

Furthermore, using $\pi_{0}$ to represent $\lambda_{2}$ which is the number of deaths per person-year in the general population, we have

$n=\frac{2}{\pi_{0}({\sqrt{IRR}- 1)}^{2}{}}$ (1)

Where n is the person-year needed, $\pi_{0}$ is the number of deaths per person-year in the general population. The component IRR is the incidence rate ratio, which is essentially the SMR for our study, given that it is age and sex-matched. Using the previously found figure of IRR = SMR = 5.86 by Arcelus and recognising $n\times\pi_{0}$ as the expected number of deaths ($e)$ in the study cohort if they have the same death rate as the general population, we can see that for a study to have an adequate sample size, it is necessary and sufficient that the expected death, $e = n\times\pi_{0} \geq\frac{2}{({\sqrt{5.86}- 1)}^{2}{}}\approx1$ .

## 5. Assessing the Certainty of Evidence

The certainty of the primary meta-analysis of 22 study units for all-cause SMR was analysed using the GRADE approach recommended by the Cochrane Handbook and required by the PRISMA 2020 statement (Higgins & Cochrane Collaboration, 2020; Page et al., 2021). There were 30,384 AN patients' data involved. Since this meta-analysis used observational studies, the initial certainty was considered to be low. For the risk of bias domain, the AHRQ grading of the NOS study quality rating showed that eight studies had good quality, nine had fair quality, and five had low quality. The overall risk of bias was considered to be of “some concern”. Most of the studies with fair quality lost scores in the selection section due to the sample not being representative of the community and choosing national mortality data instead of regional data as a comparison. This bias means our results likely only apply to AN patients receiving specialist service, but are not likely to lower our confidence in the estimate of effect. For the unexplained heterogeneity domain, substantial heterogeneity was observed despite extensive analysis. Therefore, the certainty should be downgraded. For the indirectness of the evidence domain, the AN sample mortality was directly compared to the general population mortality in the studies included.

For the imprecision of the results domain, the sample size was adequate for detecting a significant SMR in 15 out of 22 studies. Therefore, this meta-analysis, pooling all studies, has an adequate sample size. The confidence interval of the result was well above 1. For the publication bias domain, no bias was identified for our meta-analysis. On the other hand, we find a large SMR for AN patients, 5.06, with a 95% CI [3.47-7.38], and hence warrant an upgrade of certainty. In conclusion, the overall certainty of evidence was low according to the GRADE framework.

## 6. Multiple Reports of Selected Studies and Overlapping Studies

*Studies from Database Searches that are Multiple Reports of or Overlapped With the Included Studies.*

| Included studies | Multiple reports excluded | Partial overlapping studies included | Comments |
| --- | --- | --- | --- |
| (Fichter & Quadflieg, 2016) | (Fichter et al., 2003, 2006, 2017, 2021; Fichter & Quadflieg, 1999; Koch et al., 2013; Quadflieg et al., 2024) | (Quadflieg et al., 2019) | In the Quadflieg et al. (2019) study on male, 27 out of 157 of the sample were included in Fichter & Quadflieg’s (2016) study. Both studies were included in the systematic review but Quadflieg et al. (2019) were excluded for the primary meta-analysis. |
| (Nielsen & Vilmar, 2021) | (Emborg, 1999; Møller-Madsen et al., 1996; Nielsen et al., 2002) | (Joergensen, 1992; Pagsberg & Wang, 1994; Winkler et al., 2015) | All excluded studies are based on the Denmark nation-wide register data with overlapping dates, hence were considered as multiple reports of the same data. Nielsen & Vilmar (2021) reported the most updated one.  Three Denmark clinical samples were partially overlapped by Nielsen’s study. Nielsen used the Danish Psychiatric Central Research Register (PCRR), which only registered inpatient data from 1970-1995 and included outpatients. Therefore Jergensen’s (1992) and Pagsberg & Wang’s (1994) clinical sample would not be completely reported in Nielsen’s paper. Furthermore, Pagsberg & Wang’s (1994) paper used DSM as the diagnostic criteria, which differs from PCRR. Therefore, these three clinical samples were included in the systematic review. |
|  |  |  |  |
|  |  |  |  |
| Table H1 Continued | | | |
| (Franko et al., 2013) | (Blais et al., 2000; Eddy et al., 2002, 2017; Franko et al., 2018; D. B. Herzog et al., 1996, 1999, 2000; Keel et al., 2003; Keshishian et al., 2019; Thompson-Brenner et al., 2008) |  | This data from Boston had been reported extensively. |
| (Kask et al., 2016, 2017) | (Hjern et al., 2006; Kask et al., 2013; Lindblad et al., 2006; Papadopoulos et al., 2009, 2013) | (Rosling et al., 2011) | There were studies based on the Swedish national registers and Kask et al.’s (2016, 2017) are the most representative. See below for the reason for including Rosling 2011. |
| (Rosling et al., 2011) | (Norring & Sohlberg, 1993) |  | Rosling et al. (2011) was based on discharged patients from Uppsala University Hospital, with ICD-8.8 306.5.9, or ICD-9, ‘‘anorexia nervosa’’ (307B) or ‘‘other unspecified eating problems’’ (307F). Then, the diagnosis of DSM IV AN was done by clinical note review. Therefore, Rosling’s sample was not entirely included in Kask’s study (Kask et al., 2016, 2017), and Rosling provided a more homogenous clinic sample, which is therefore included in the systematic review. |
| (Himmerich et al., 2019) |  | (Hoang et al., 2014) | Himmerich recruited patients from the South London and Maudsley NHS Foundation Trust with a diagnosis received between or before 2007 and 2016. Hong recruited patients discharged from National Health Service (NHS) hospitals in England with an AN diagnosis from 2001 to 2009. There are minimal overlaps between 2007-2009. Both are retained for systematic review. |
| (Korndörfer et al., 2003) | (Iacovino, 2004; Nelson, 2003) |  |  |
| (Signorini et al., 2007) | (De Filippo et al., 2000) |  |  |
| (Speranza et al., 2020) | (Di Vincenzo O., 2018; Speranza et al., 2020) |  |  |
| (Guinhut et al., 2021) | (Guinhut et al., 2019) |  |  |
| Table H1 Continued |  |  |  |
| (Winkler et al., 2015) | (Støving et al., 2011; Winkler, 2017) |  |  |
| (H. R. Millar et al., 2005) | (H. Millar et al., 2004) |  |  |
| (A. Crisp & Collaborators, 2006) | (A. H. Crisp et al., 1992) |  |  |
| (Löwe et al., 2001) | (Deter et al., 2005; Deter & Herzog, 1994; W. Herzog, Deter, et al., 1997; W. Herzog, Schellberg, et al., 1997; Zipfel et al., 2000) |  |  |

**Reference**

Blais, M. A., Becker, A. E., Burwell, R. A., Flores, A. T., Nussbaum, K. M., Greenwood, D. N., Ekeblad, E. R., & Herzog, D. B. (2000). Pregnancy: Outcome and impact on symptomatology in a cohort of eating-disordered women. *The International Journal of Eating Disorders*, *27*(2), 140–149. https://doi.org/10.1002/(sici)1098-108x(200003)27:2<140::aid-eat2>3.0.co;2-e

Crisp, A. & Collaborators. (2006). 1.6. Death, survival and recovery in anorexia nervosa: A thirty five year study. *European Eating Disorders Review*, *14*(3), 168–175. https://doi.org/10.1002/erv.704

Crisp, A. H., Callender, J. S., Halek, C., & Hsu, L. K. (1992). Long-term mortality in anorexia nervosa. A 20-year follow-up of the St George’s and Aberdeen cohorts. *The British Journal of Psychiatry: The Journal of Mental Science*, *161*, 104–107. https://doi.org/10.1192/bjp.161.1.104

De Filippo, E., Signorini, A., Bracale, R., Pasanisi, F., & Contaldo, F. (2000). Hospital admission and mortality rates in anorexia nervosa: Experience from an integrated medical-psychiatric outpatient treatment. *Eating and Weight Disorders: EWD*, *5*(4), 211–216. https://doi.org/10.1007/BF03354448

Deter, H. C., & Herzog, W. (1994). Anorexia nervosa in a long-term perspective: Results of the Heidelberg-Mannheim Study. *Psychosomatic Medicine*, *56*(1), 20–27. https://doi.org/10.1097/00006842-199401000-00003

Deter, H. C., Schellberg, D., Köpp, W., Friederich, H. C., & Herzog, W. (2005). Predictability of a favorable outcome in anorexia nervosa. *European Psychiatry: The Journal of the Association of European Psychiatrists*, *20*(2), 165–172. https://doi.org/10.1016/j.eurpsy.2004.09.006

Di Vincenzo O., S. E. (2018). *Long term mortality in Anorexia Nervosa: Results of a 10-year follow-up and a an updated review of the literature. Obesity Facts, 11(Supplement 1), 119. , Santarpia L., De Filippo E., De Caprio C., Sammarco R., , et al (2018). ECO 2018*, 119. https://doi.org/10.1159/000489691

Eddy, K. T., Keel, P. K., Dorer, D. J., Delinsky, S. S., Franko, D. L., & Herzog, D. B. (2002). Longitudinal comparison of anorexia nervosa subtypes. *The International Journal of Eating Disorders*, *31*(2), 191–201. https://doi.org/10.1002/eat.10016

Eddy, K. T., Tabri, N., Thomas, J. J., Murray, H. B., Keshaviah, A., Hastings, E., Edkins, K., Krishna, M., Herzog, D. B., Keel, P. K., & Franko, D. L. (2017). Recovery From Anorexia Nervosa and Bulimia Nervosa at 22-Year Follow-Up. *The Journal of Clinical Psychiatry*, *78*(2), 184–189. https://doi.org/10.4088/JCP.15m10393

Emborg, C. (1999). Mortality and causes of death in eating disorders in Denmark 1970-1993: A case register study. *The International Journal of Eating Disorders*, *25*(3), 243–251. https://doi.org/10.1002/(sici)1098-108x(199904)25:3<243::aid-eat1>3.0.co;2-2

Fichter, M. M., Naab, S., Voderholzer, U., & Quadflieg, N. (2021). Mortality in males as compared to females treated for an eating disorder: A large prospective controlled study. *Eating and Weight Disorders: EWD*, *26*(5), 1627–1637. https://doi.org/10.1007/s40519-020-00960-1

Fichter, M. M., & Quadflieg, N. (1999). Six-year course and outcome of anorexia nervosa. *The International Journal of Eating Disorders*, *26*(4), 359–385. https://doi.org/10.1002/(sici)1098-108x(199912)26:4<359::aid-eat2>3.0.co;2-7

Fichter, M. M., & Quadflieg, N. (2016). Mortality in eating disorders—Results of a large prospective clinical longitudinal study. *The International Journal of Eating Disorders*, *49*(4), 391–401. https://doi.org/10.1002/eat.22501

Fichter, M. M., Quadflieg, N., Crosby, R. D., & Koch, S. (2017). Long-term outcome of anorexia nervosa: Results from a large clinical longitudinal study. *The International Journal of Eating Disorders*, *50*(9), 1018–1030. https://doi.org/10.1002/eat.22736

Fichter, M. M., Quadflieg, N., & Hedlund, S. (2006). Twelve-year course and outcome predictors of anorexia nervosa. *The International Journal of Eating Disorders*, *39*(2), 87–100. https://doi.org/10.1002/eat.20215

Fichter, M. M., Quadflieg, N., & Rehm, J. (2003). Predicting the outcome of eating disorders using structural equation modeling. *The International Journal of Eating Disorders*, *34*(3), 292–313. https://doi.org/10.1002/eat.10193

Franko, D. L., Keshaviah, A., Eddy, K. T., Krishna, M., Davis, M. C., Keel, P. K., & Herzog, D. B. (2013). A longitudinal investigation of mortality in anorexia nervosa and bulimia nervosa. *The American Journal of Psychiatry*, *170*(8), 917–925. https://doi.org/10.1176/appi.ajp.2013.12070868

Franko, D. L., Tabri, N., Keshaviah, A., Murray, H. B., Herzog, D. B., Thomas, J. J., Coniglio, K., Keel, P. K., & Eddy, K. T. (2018). Predictors of long-term recovery in anorexia nervosa and bulimia nervosa: Data from a 22-year longitudinal study. *Journal of Psychiatric Research*, *96*, 183–188. https://doi.org/10.1016/j.jpsychires.2017.10.008

Guinhut, M., Godart, N., Benadjaoud, M.-A., Melchior, J.-C., & Hanachi, M. (2021). Five-year mortality of severely malnourished patients with chronic anorexia nervosa admitted to a medical unit. *Acta Psychiatrica Scandinavica*, *143*(2), 130–140. https://doi.org/10.1111/acps.13261

Guinhut, M., Melchior, J.-C., Benadjaoud, M., Godart, N., & Mouna, H. (2019). OR45: 5-Year Mortality of 384 Severely Malnourished Anorexia Nervosa (AN) Adult Patients After Admission in a Clinical-Nutrition-Unit. *Clinical Nutrition*, *38*, S21–S22. https://doi.org/10.1016/S0261-5614(19)32517-8

Herzog, D. B., Dorer, D. J., Keel, P. K., Selwyn, S. E., Ekeblad, E. R., Flores, A. T., Greenwood, D. N., Burwell, R. A., & Keller, M. B. (1999). Recovery and relapse in anorexia and bulimia nervosa: A 7.5-year follow-up study. *Journal of the American Academy of Child and Adolescent Psychiatry*, *38*(7), 829–837. https://doi.org/10.1097/00004583-199907000-00012

Herzog, D. B., Field, A. E., Keller, M. B., West, J. C., Robbins, W. M., Staley, J., & Colditz, G. A. (1996). Subtyping eating disorders: Is it justified? *Journal of the American Academy of Child and Adolescent Psychiatry*, *35*(7), 928–936. https://doi.org/10.1097/00004583-199607000-00020

Herzog, D. B., Greenwood, D. N., Dorer, D. J., Flores, A. T., Ekeblad, E. R., Richards, A., Blais, M. A., & Keller, M. B. (2000). Mortality in eating disorders: A descriptive study. *The International Journal of Eating Disorders*, *28*(1), 20–26. https://doi.org/10.1002/(sici)1098-108x(200007)28:1<20::aid-eat3>3.0.co;2-x

Herzog, W., Deter, H. C., Fiehn, W., & Petzold, E. (1997). Medical findings and predictors of long-term physical outcome in anorexia nervosa: A prospective, 12-year follow-up study. *Psychological Medicine*, *27*(2), 269–279. https://doi.org/10.1017/s0033291796004394

Herzog, W., Schellberg, D., & Deter, H. C. (1997). First recovery in anorexia nervosa patients in the long-term course: A discrete-time survival analysis. *Journal of Consulting and Clinical Psychology*, *65*(1), 169–177. https://doi.org/10.1037//0022-006x.65.1.169

Himmerich, H., Hotopf, M., Shetty, H., Schmidt, U., Treasure, J., Hayes, R. D., Stewart, R., & Chang, C.-K. (2019). Psychiatric comorbidity as a risk factor for mortality in people with anorexia nervosa. *European Archives of Psychiatry and Clinical Neuroscience*, *269*(3), 351–359. https://doi.org/10.1007/s00406-018-0937-8

Hjern, A., Lindberg, L., & Lindblad, F. (2006). Outcome and prognostic factors for adolescent female in-patients with anorexia nervosa: 9- to 14-year follow-up. *The British Journal of Psychiatry: The Journal of Mental Science*, *189*, 428–432. https://doi.org/10.1192/bjp.bp.105.018820

Hoang, U., Goldacre, M., & James, A. (2014). Mortality following hospital discharge with a diagnosis of eating disorder: National record linkage study, England, 2001-2009. *The International Journal of Eating Disorders*, *47*(5), 507–515. https://doi.org/10.1002/eat.22249

Iacovino, J. R. (2004). Anorexia nervosa: A 63-year population-based survival study. *Journal of Insurance Medicine (New York, N.Y.)*, *36*(2), 107–110.

Joergensen, J. (1992). The epidemiology of eating disorders in Fyn County, Denmark, 1977-1986. *Acta Psychiatrica Scandinavica*, *85*(1), 30–34. https://doi.org/10.1111/j.1600-0447.1992.tb01438.x

Kask, J., Ekselius, L., Brandt, L., Ekbom, A., & Papadopoulos, F. C. (2013). 2138 – Psychiatric comorbidity and mortality in women with anorexia nervosa. *European Psychiatry*, *28*, 1. https://doi.org/10.1016/S0924-9338(13)77023-8

Kask, J., Ekselius, L., Brandt, L., Kollia, N., Ekbom, A., & Papadopoulos, F. C. (2016). Mortality in Women With Anorexia Nervosa: The Role of Comorbid Psychiatric Disorders. *Psychosomatic Medicine*, *78*(8), 910–919. https://doi.org/10.1097/PSY.0000000000000342

Kask, J., Ramklint, M., Kolia, N., Panagiotakos, D., Ekbom, A., Ekselius, L., & Papadopoulos, F. C. (2017). Anorexia nervosa in males: Excess mortality and psychiatric co-morbidity in 609 Swedish in-patients. *Psychological Medicine*, *47*(8), 1489–1499. https://doi.org/10.1017/S0033291717000034

Keel, P. K., Dorer, D. J., Eddy, K. T., Franko, D., Charatan, D. L., & Herzog, D. B. (2003). Predictors of mortality in eating disorders. *Archives of General Psychiatry*, *60*(2), 179–183. https://doi.org/10.1001/archpsyc.60.2.179

Keshishian, A. C., Tabri, N., Becker, K. R., Franko, D. L., Herzog, D. B., Thomas, J. J., & Eddy, K. T. (2019). Eating disorder recovery is associated with absence of major depressive disorder and substance use disorders at 22-year longitudinal follow-up. *Comprehensive Psychiatry*, *90*, 49–51. https://doi.org/10.1016/j.comppsych.2019.01.002

Koch, S., Quadflieg, N., & Fichter, M. (2013). Purging disorder: A comparison to established eating disorders with purging behaviour. *European Eating Disorders Review: The Journal of the Eating Disorders Association*, *21*(4), 265–275. https://doi.org/10.1002/erv.2231

Korndörfer, S. R., Lucas, A. R., Suman, V. J., Crowson, C. S., Krahn, L. E., & Melton, L. J. (2003). Long-term survival of patients with anorexia nervosa: A population-based study in Rochester, Minn. *Mayo Clinic Proceedings*, *78*(3), 278–284. https://doi.org/10.4065/78.3.278

Lindblad, F., Lindberg, L., & Hjern, A. (2006). Improved survival in adolescent patients with anorexia nervosa: A comparison of two Swedish national cohorts of female inpatients. *The American Journal of Psychiatry*, *163*(8), 1433–1435. https://doi.org/10.1176/ajp.2006.163.8.1433

Löwe, B., Zipfel, S., Buchholz, C., Dupont, Y., Reas, D. L., & Herzog, W. (2001). Long-term outcome of anorexia nervosa in a prospective 21-year follow-up study. *Psychological Medicine*, *31*(5), 881–890. https://doi.org/10.1017/s003329170100407x

Millar, H. R., Wardell, F., Vyvyan, J. P., Naji, S. A., Prescott, G. J., & Eagles, J. M. (2005). Anorexia nervosa mortality in Northeast Scotland, 1965-1999. *The American Journal of Psychiatry*, *162*(4), 753–757. https://doi.org/10.1176/appi.ajp.162.4.753

Millar, H., Wardell, F., Naji, S., Vyvyan, J., & Eagles, J. (2004). Mortality in anorexia nervosa in Northeast Scotland. *International Journal of Eating Disorders*, *35*, 387–387.

Møller-Madsen, S., Nystrup, J., & Nielsen, S. (1996). Mortality in anorexia nervosa in Denmark during the period 1970-1987. *Acta Psychiatrica Scandinavica*, *94*(6), 454–459. https://doi.org/10.1111/j.1600-0447.1996.tb09890.x

Nelson, R. A. (2003). Long-term survival of patients with anorexia nervosa. *Mayo Clinic Proceedings*, *78*(5), 655; author reply 655. https://doi.org/10.4065/78.5.655

Nielsen, S., Emborg, C., & Mølbak, A.-G. (2002). Mortality in concurrent type 1 diabetes and anorexia nervosa. *Diabetes Care*, *25*(2), 309–312. https://doi.org/10.2337/diacare.25.2.309

Nielsen, S., & Vilmar, J. W. (2021). What can we learn about eating disorder mortality from eating disorder diagnoses at initial assessment? A Danish nationwide register follow-up study using record linkage, encompassing 45 years (1970-2014). *Psychiatry Research*, *303*, 114091. https://doi.org/10.1016/j.psychres.2021.114091

Norring, C. E., & Sohlberg, S. S. (1993). Outcome, recovery, relapse and mortality across six years in patients with clinical eating disorders. *Acta Psychiatrica Scandinavica*, *87*(6), 437–444. https://doi.org/10.1111/j.1600-0447.1993.tb03401.x

Pagsberg, A. K., & Wang, A. R. (1994). Epidemiology of anorexia nervosa and bulimia nervosa in Bornholm County, Denmark, 1970-1989. *Acta Psychiatrica Scandinavica*, *90*(4), 259–265. https://doi.org/10.1111/j.1600-0447.1994.tb01590.x

Papadopoulos, F. C., Ekbom, A., Brandt, L., & Ekselius, L. (2009). Excess mortality, causes of death and prognostic factors in anorexia nervosa. *The British Journal of Psychiatry: The Journal of Mental Science*, *194*(1), 10–17. https://doi.org/10.1192/bjp.bp.108.054742

Papadopoulos, F. C., Karamanis, G., Brandt, L., Ekbom, A., & Ekselius, L. (2013). Childbearing and mortality among women with anorexia nervosa. *The International Journal of Eating Disorders*, *46*(2), 164–170. https://doi.org/10.1002/eat.22051

Quadflieg, N., Naab, S., Fichter, M., & Voderholzer, U. (2024). Long-Term Outcome and Mortality in Adolescent Girls 8 Years After Treatment for Anorexia Nervosa. *International Journal of Eating Disorders*, *57*(12), 2497–2503. https://doi.org/10.1002/eat.24299

Quadflieg, N., Strobel, C., Naab, S., Voderholzer, U., & Fichter, M. M. (2019). Mortality in males treated for an eating disorder-A large prospective study. *The International Journal of Eating Disorders*, *52*(12), 1365–1369. https://doi.org/10.1002/eat.23135

Rosling, A. M., Sparén, P., Norring, C., & von Knorring, A.-L. (2011). Mortality of eating disorders: A follow-up study of treatment in a specialist unit 1974-2000. *The International Journal of Eating Disorders*, *44*(4), 304–310. https://doi.org/10.1002/eat.20827

Signorini, A., De Filippo, E., Panico, S., De Caprio, C., Pasanisi, F., & Contaldo, F. (2007). Long-term mortality in anorexia nervosa: A report after an 8-year follow-up and a review of the most recent literature. *European Journal of Clinical Nutrition*, *61*(1), 119–122. https://doi.org/10.1038/sj.ejcn.1602491

Speranza, E., Santarpia, L., Marra, M., De Filippo, E., Di Vincenzo, O., Morlino, D., Pasanisi, F., & Contaldo, F. (2020). Long-Term Outcomes from a 10-Year Follow-Up of Women Living with a Restrictive Eating Disorder: A Brief Report. *Nutrients*, *12*(8), 2331. https://doi.org/10.3390/nu12082331

Støving, R. K., Andries, A., Brixen, K., Bilenberg, N., & Hørder, K. (2011). Gender differences in outcome of eating disorders: A retrospective cohort study. *Psychiatry Research*, *186*(2–3), 362–366. https://doi.org/10.1016/j.psychres.2010.08.005

Thompson-Brenner, H., Eddy, K. T., Franko, D. L., Dorer, D. J., Vashchenko, M., Kass, A. E., & Herzog, D. B. (2008). A personality classification system for eating disorders: A longitudinal study. *Comprehensive Psychiatry*, *49*(6), 551–560. https://doi.org/10.1016/j.comppsych.2008.04.002

Winkler, L. A.-D. (2017). Funen Anorexia Nervosa Study—A follow-up study on outcome, mortality, quality of life and body composition. *Danish Medical Journal*, *64*(6), B5380.

Winkler, L. A.-D., Bilenberg, N., Hørder, K., & Støving, R. K. (2015). Does specialization of treatment influence mortality in eating disorders?—A comparison of two retrospective cohorts. *Psychiatry Research*, *230*(2), 165–171. https://doi.org/10.1016/j.psychres.2015.08.032

Zipfel, S., Löwe, B., Reas, D. L., Deter, H. C., & Herzog, W. (2000). Long-term prognosis in anorexia nervosa: Lessons from a 21-year follow-up study. *Lancet (London, England)*, *355*(9205), 721–722. https://doi.org/10.1016/S0140-6736(99)05363-5

## 7. Non-English Studies excluded

## Alvin P., Zogheib J., Rey C., & Losay J. (1993). Severe complications of eating disorders in adolescents. Archives Francaises de Pediatrie, 50(9), 755–762.

Background: Subclinical medical complications frequently occur during the follow-up of anorexia nervosa and bulimia. This paper describes some of these. Population and methods: Charts of 99 adolescent patients (89 girls and 10 boys), aged 11.8 to 22 years (mean: 16.6 +/- 2.1 years), admitted for anorexia nervosa (N : 92) or bulimia (N : 7), were analyzed retrospectively. All severe or potentially severe, clinical and non-clinical, findings at admission were included in the study. Result(s): Anorexic patients had a mean weight loss of 31.5% (22 of them were also vomiters or laxative abusers). Initial nasogastric tube feeding was necessary in 19 patients and parenteral nutrition in 2. Bradycardia and hypotension were common. A variety of ECG abnormalities were seen in 86% of the patients. Mitral valve prolapse was present in 14 of the 43 patients examined by echocardiography. Electrolyte imbalance was also common: hyponatremia in 7 patients, hypokalemia in 21, hypochloremia in 10 of the 12 vomiters, hypophosphatemia in 7, hyperazotemia in 24 and hypoglycemia in 22. Bone marrow hypoplasia was frequent, with leukopenia in 29 patients, anemia in 21 and thrombocytopenia in 5. No patient developed infectious complications. One patient presented with an acute gastric dilatation and another with spontaneous pneumomediastinum. One patient, 14 year-old, died 3 years after the onset of anorexia from acute water intoxication. Conclusion(s): These well-known complications are more common in anorexic than in bulimic patients. Their prevention required rigorous and continuous medical supervision.

## Deter, H. C., Herzog, W., & Manz, R. (1994). [Do patients with anorexia nervosa return to psychological health? Results of a 12-year follow-up of 103 patients]. Werden Anorexia Nervosa-Patienten Seelisch Wieder Gesund? Ergebnisse Einer 12-Jahreskatamnese von 103 Patienten., 40(2), 155–173.

In a long-term follow-up study over 12.7% (range 9-19) years, 16 of 103 patients with anorexia nervosa died. 92% of the survivors were followed up personally, 7% only indirectly. The paper presents the psychological changes assessed by means of the Anorexia Nervosa Symptom Score in the course of the disorder. At the time of catamnesis, the psychic condition of the patients was furthermore evaluated in more detail by procedures of independent assessment (DSM III-R, Impairment-Score (IS), Confidant Rating) as well as by using different instruments of self-assessment (Eating Attitude Test, Eating Disorder Inventory, Social Network Questionnaire). In a case conference taking into account all relevant findings, 40.1% of the patients were found to be healthy, 30.4% were considered to still show severe psychic disorders, and 29.4% were rated as desolate. These results suggest that part of the patients become psychicalls healthy again. Altogether, however, the long-term prognosis of anorexia has to be regarded as rather poor.

## Deter, H.-C., Köpp, W., Zipfel, S., & Herzog, W. (1998). Männliche anorexia-nervosa-patienten im langzeitverlauf. Der Nervenarzt, 69(5), 419–426. https://doi.org/10.1007/s001150050291

Investigated possible differences in the disease course of male and female patients with anorexia nervosa. Total assessment after a mean of 12 yrs revealed a clearly increased mortality rate of more than 20% in 10 of the male patients examined (as well as 2 male anorexia nervosa patients with an additional physical condition). The male survivors had a better prognosis than the female patients. Male anorectics were also in better physical condition than their female counterparts at the time of follow-up. On the Morgan Russell scale, food intake, psychosexual state and social state showed a more favorable development in male than in female patients. Male patients had a more favorable course regarding psychosocial integration but a similar course to female patients regarding eating disorder symptoms. (PsycINFO Database Record (c) 2016 APA, all rights reserved)

## Fichter M.M., Quadflieg N., & Lindner S. (2014). Internet-based relapse prevention in anorexia nervosa—Long-term course in an intent-to-treat sample. Zeitschrift fur Psychiatrie, Psychologie und Psychotherapie, 62(1), 35–42. https://doi.org/10.1024/1661-4747/a000176

Anorexia nervosa is a mental disorder associated with high premature mortality in females. While the efficacy of psychotherapy for anorexia nervosa is well documented, little is known regarding relapse prevention following psychotherapy. The present study reports on a randomised controlled study of internet-based relapse prevention over nine months after inpatient treatment for anorexia nervosa and an additional nine months of follow-up. A large sample of 258 female patients was randomised to one of two treatment conditions (relapse prevention or controls). Intent-to-treat analyses over three assessment points (beginning and end of intervention, follow-up) were conducted. Primary outcome was body weight. Patients completing all nine modules of the program showed significantly more weight gain during intervention and in the nine months following the end of intervention. Course of weight in patients who completed less than all nine modules of the program did not differ significantly from controls. Controls showed a small decrease of weight during the intervention and a weight gain after the end of intervention till follow-up. Most patients reported the program as recommendable and helpful. The results of this study confirm the feasibility of internet-based relapse prevention in anorexia nervosa. © 2014 Verlag Hans Huber, Hogrefe AG.

## Garcia de Amusquibar, A. M., Matusevich, D., Gutt, S., de la Parra, I., Girard, P., & Finkelsztein, C. (2005). [Hospitalization of patients with eating disorders: An eight years review]. Experiencia de Ocho Anos de Internacion Con Trastornos de La Conducta Alimentaria., 16(64), 405–411.

Eating disorders (Eds) are pathologies with a multifactorial etiopathogenesis. Their treatment requires an interdisciplinary team. The aim of this paper is to discuss in patient treatment (n=80) of patients with eating disorders during eight years. This treatment was in charge of the Eating Disorders Team of the Hospital Italiano of Buenos Aires. The approach to critical stages of eating disorders envolves the clinical or psychiatric admission of the patients as the principal means to ward off patients from death or the appearance of cronifications. 77,5% of the patients were admitted to the psychiatric ward, 5% to a clinical ward and 17,5% to a clinical followed by a psychiatric ward. The average B.M.I. in admission was 16,83. The average time in psychiatry was 35 days. During inpatient treatment 21,25% required a nasogastric tube and 82% received psychopharmacological treatment. The mortality rate was 1,25%. During the ambulatory treatment that followed hospitalizations, 32,5% were readmitted to the psychiatric ward and there were 10% of dropouts. Hospitalization of these patients looks after the achievement of healthier eating habits and the establishment of a therapeutic alliance which allows the follow up of an ambulatory treatment.

## Godart N., Duclos J., Hubert T., Carrot B., Chapelon E., Roux H., Ali A., & Jeammet P. (2014). Hospitalization for anorexia nervosa during adolescence: What happens 10years later for those young women? Neuropsychiatrie de l’Enfance et de l’Adolescence, 62(5), 263–270. https://doi.org/10.1016/j.neurenf.2014.05.004

Objectives: Recent studies in Sweden have shown that the mortality observed in recent years following hospitalisation for anorexia nervosa (AN) is falling. A study carried out by our team in 1991 showed a fairly high mortality rate of 7%, 11years after hospitalisation for AN. What is the situation in 2011? What is the outcome of patients who have followed a care programme that has undergone changes? Patients and Methods: The study reconstituted a cohort of 180 patients hospitalised for AN between the age of 13 and 22years. It was possible to evaluate 180young women with regard to vital status, on average 9.7+/-1.6years after their hospitalisation. Ninety-seven young women (51%) were also evaluated with respect to their outcomes-psychological, eating habits, physical and social conditions. This was done using a self-assessment (clinical questionnaire, the Eating Disorder Inventory [EDI-2], the Social Adjustment Scale, Self-Report [SAS-SR] and the World Health Organization Questionnaire Quality of Life [WHOQOL short-form]) and a face-to-face interview looking for clinical data using questionnaires and structured instruments (Mini International Neuropsychiatric Interview [MINI], Morgan and Russell outcome scale). Result(s): Among the 180 subjects in the initial sample, four young women had died, i.e. 2.1% of the sample. The 97subjects who were re-assessed were not different from the others at the time of hospitalisation. In the follow-up evaluation, the overall outcome of the patients was good or intermediate for 62.9% of the subjects. Their actual BMI was variable, ranging from underweight (10.94. kg/m) to overweight (27.01. kg/m), with a mean of 19.38. +/-. 2.71. kg/m. BMI was above the AN threshold for 85.3% of cases, and menstruation was present in about the same proportion. The mean score on the EDI-2 was fairly good, although it was 10 points above the mean score for the general population (54.94. +/-. 40.05) compared to 44 in the general population; this is nevertheless well above the mean for an anorexic population, which is 106. Only 15.5% of the subjects (n= 15) were still suffering from AN or bulimia (BN) according to complete DSM-IV criteria. Four were AN-R, and 11 were alternating from AN to BN. Social adjustment was good on average (mean score of 1.74. +/-. 0.43 on SAS-SR scale) as was their quality of life (mean score 67.05. +/-. 18.37 on the WHOQOL short form). The mortality of our sample was six times over the rate observed in general population. Conclusion(s): AN that requires hospitalization during adolescence is a high morbi-mortality pathology. © 2014 Elsevier Masson SAS.

## Herzog T., Hartmann A., & Falk C. (1996). The short-term effects of psychodynamic inpatient treatment of anorexia nervosa with and without an explicit focus on eating pathology—A controlled study. PPmP Psychotherapie Psychosomatik Medizinische Psychologie, 46(1), 11–22.

Inpatient treatment programs for severely anorectic patients often combine or integrate psychodynamic approaches with an explicit focus on symptoms. There is a dearth of studies evaluating treatments, let alone in a controlled way. In a quasi-experimental design we compare the normalization of body weight in the 20 consecutive admission episodes before ('PD’) and the 20 episodes after ('PD and SY’) introduction and integration of explicit symptom orientation derived from the St. Georges model (Crisp 1980) on a psycho-analytically oriented psychosomatic ward. All patients fulfill ICD-10 criteria for either restrictive (F50.00) or bulimic (F50.01) anorexia nervosa. In addition to AN(C)OVAS growth curve modelling and multi state analysis, a variant of survival analysis, are used to model the shape and temporal course of treatment responses. Clinical features at admission and total duration of treatment and drop-out rates are comparable. The PD and SY group reaches target weight significantly more frequently (14 vs. 5 episodes, p = 0,023) and with a much higher total probability (0,70 vs. 0.25 in the PD group, Cox regression p = 0.002). After a phase of adaptation the treatment team feels relieved by the more systematically structured approach and is now able to treat a far larger number of patients. It does not seem justifiable anymore to withhold anorectic patients explicitely symptom-oriented treatment components. These and other conclusions for clinical practice and research are discussed.

## Herzog, T., Hartmann, A., & Falk, C. (1996). [Total symptom-oriented and psychodynamic concept in inpatient treatment of anorexia nervosa. A quasi-experimental comparative study of 40 admission episodes]. Symptomorientierung Und Psychodynamisches Gesamtkonzept Bei Der Stationaren Behandlung Der Anorexia Nervosa. Eine Quasi-Experimentelle Vergleichsuntersuchung von 40 Aufnahmeepisoden., 46(1), 11–22.

Inpatient treatment programs for severely anorectic patients often combine or integrate psychodynamic approaches with an explicit focus on symptoms. There is a dearth of studies evaluating treatments, let alone in a controlled way. In a quasi-experimental design we compare the normalization of body weight in the 20 consecutive admission episodes before (“PD”) and the 20 episodes after (“PD & SY”) introduction and integration of explicit symptom orientation derived from the St. Georges model (Crisp 1980) on a psychoanalytically oriented psychosomatic ward. All patients fulfill ICD-10 criteria for either restrictive (F50.00) or bulimic (F50.01) anorexia nervosa. In addition to AN(C)OVAS growth curve modelling and multi state analysis, a variant of survival analysis, are used to model the shape and temporal course of treatment responses. Clinical features at admission and total duration of treatment and drop-out rates are comparable. The PD & SY group reaches target weight significantly more frequently (14 vs. 5 episodes, p = 0.023) and with a much higher total probability (0.70 vs. 0.25 in the PD group, Cox regression p = 0.002). After a phase of adaptation the treatment team feels relieved by the more systematically structured approach and is now able to treat a far larger number of patients. It does not seem justifiable anymore to withhold anorectic patients explicitely symptom-oriented treatment components. These and other conclusions for clinical practice and research are discussed.

## Hjalmers, I. (1996). [Anorexia nervosa—A severe somatic and psychiatric diagnosis! One does not die of anxiety but of caloric deficiency]. Anorexia Nervosa--Allvarlig Somatisk Och Psykiatrisk Diagnos! Man Dor Inte Av Angest Utan Av Kaloribrist., 93(26), 2514–2515.

Abstract not available.

## Jeammet, P., Brechon, G., Payan, C., Gorge, A., & Fermanian, J. (1991). [The outcome of anorexia nervosa: A prospective study of 129 patients evaluated at least 4 years after their first admission]. Le Devenir de l’anorexie Mentale: Une Etude Prospective de 129 Patients Evalues Au Moins 4 Ans Apres Leur Premiere Admission., 34(2), 381–442.

This is a follow-up study with an interval of 4 to 20 years (mean: 11 years) of 146 cases of anorexia nervosa of which 129 could be contacted. The investigation is based on various measures: BPRS scale, HSCL, a chart of clinical assessment made by the examiner (10 items), a global self-evaluation by the patient, a clinical interview. Results and factors of prognosis are discussed. Our findings stress the importance of good outcome on a long term basis but also the potential severity of anorexia nervosa with both a death risk and a risk of chronicity. It becomes obvious that the major challenge of anorectic conduct are the residual disturbances of the personality. These disturbances are mainly difficulties in investing and the antagonism between the objectal inclination of these patients and the need to protect their narcissistic balance. A posteriori we understand the defensive meaning of the anorectic conduct and its value as a reorganizing of objectal relations. The psychopathological significance of this conduct, its stakes and its therapeutical consequences are discussed.

## Jonsson, P. H. (2001). [A study of patients with anorexia nervosa in Gavleborg. More boys than girls required intensive care]. Studie i Gavleborg Av Patienter Med Anorexia Nervosa. Fler Pojkar an Flickor Intensivvardas., 98(42), 4578–4582.

In the early 1970’s British doctors (Beaumont et al) published clinical findings and concluded that anorexia nervosa is a disease occurring in both sexes. Until then it had been considered a female disease only. A number of studies have reported a rather poor outcome for boys and men. In a Danish study infertility was noted in affected men. The aim of this study is to describe typical arrays of clinical data in order to assess attitudes and values concerning the effect of treatment on 28 teenagers out of a total of 48 children and adolescents. A sociometric questionnaire was used to assess the youngsters’ knowledge of their disease, their opinions concerning the competence of staff, their feelings concerning treatment, including parental involvement in therapy and medications used, as well as their opinions concerning the results of treatment. All boys are alive (in 1999), 19 years after falling ill. The boys were much more reluctant to accept inpatient care than were the girls, who seem to show greater autonomy. Participants ’ final appraisal of treatment is slightly positive, whereas in the hypothetical situation that a friend might fall ill with an eating disorder, a sizeable majority would recommend contact with child psychiatry.

## Kachele, H. (1999). [A multicenter study of expenditure and success in psychodynamic therapy of eating disorders. Study design and initial results]. Eine Multizentrische Studie Zu Aufwand Und Erfolg Bei Psychodynamischer Therapie von Essstorungen. Studiendesign Und Erste Ergebnisse. Studiengruppe MZ-ESS., 49(3), 100–108.

Evaluation of psychodynamically oriented treatment of anorectic and bulimic patients, taking into account both quality and cost of therapist (“How much therapy is applied to which patients for a successful treatment?”). Criteria for indication of the kind of therapy and of amount of therapy (“dosage”) are provided., METHOD: Prospective naturalistic longitudinal study (comparable to phase IV of effectiveness studies on pharmaceuticals) including 2.5 year follow-up. Consecutive sample (Anorexia nervosa and bulimia nervosa, DSM-III-R). Multidimensional outcome measures including self-rating and expert-ratings. Operationalisation of outcome is layered. Differentiated measurement of amount of therapy and treatment elements. Survival analysis, logistic regression., RESULTS: 1171 completely documented treatment episodes of 43 institutions (30.3% Anorexia nervosa, 55.3% bulimia nervosa, 14.4% double diagnoses). Patients are chronically ill (M = 7.6 years; SD = 6.3 years) and at the beginning of (mostly inpatient) treatment of the index episode 25.5 years of age (SD = 6.0 years). Therapies last appr. 11 weeks (median) and encompass a broad range of therapy measures., DISCUSSION: Study conduction as well as data collection and analysis of this worldwide largest study on treatment of eating disorders is discussed by presenting preliminary results.

## Moller-Madsen, S. M., Nystrup, J., & Nielsen, S. (1998). [Mortality of anorexia nervosa in Denmark 1970-1987]. Dodeligheden Af Anorexia Nervosa i Danmark 1970-1987., 160(38), 5509–5513.

Eight hundred and fifty-three patients were admitted to psychiatric institutions in Denmark with anorexia nervosa between 1970 and 1986. Based on register information, 50 deaths were recorded during a mean follow-up period of 7.8 years. Amongst these, five were males and 45 females. The standardized mortality ratio (SMR) was 9.1 in both sexes. The SMR was maximal during the first year after index admission. Suicide was the dominant cause of death amongst subjects who died from unnatural causes (18 of 22 cases). Among those who died from natural causes (24 subjects), 13 individuals died from anorexia nervosa, and 11 individuals died from other illnesses.

## Nakayama, T., & Nozoe, S. (2001). [Anorexia nervosa (AN)—Epidemiology, cause, therapy, outcome]. Nihon Rinsho. Japanese Journal of Clinical Medicine, 59(3), 534–539.

The number of patients suffering from anorexia nervosa is rising steadily. More than 90 percent of patients are female in preadolescents or adolescents. AN is characterized by abnormal eating behavior and excessive loss of weight. The self-esteem is highly dependent on their body shape and weight. The purposes of behavior therapy for AN are to remove various types of avoidance behavior, to reestablish desirable eating behavior and social skill behavior. The course and outcome of AN are variable. Of patients admitted to our hospital to receive behavior therapy, 60 percent are mostly recovered, 30 percent exhibit a fluctuating pattern of weight gain followed by relapse, and 10 percent are chronically deteriorating course of the illness over many years. The long-term mortality from AN is about 6 percent.

## Rabe-Jablonska J. (2003). Anorexia nervosa in adult women. Psychiatria Polska, 37(1), 29–37.

Aim and method: The autor presents the clinical picture of anorexia nervosa and comorbidity in women with serious symptoms of anorexia nervosa (diagnosed acc. to DSM-IV), which appeared in adulthood, after the age of 25 years. Result(s): Most of these patients (25-40 years old) had mild symptoms of eating disorders (restricted anorexia nervosa), from adolescence, never diagnosed and treated, had a nondisturbed somatic state and social functioning. The worsening of psychic state and full development of anorexia nervosa symptoms occurred during stressful life event (eg. avoidance by sexual partner). The majority of those patients had present and past (from adolescence) various anxiety and depressive disorders and personality disorders (obsessive - compulsive, borderline). In most of the women older than 40 years, anorexia nervosa comorbided with depressive disorders, less often with mixed, anxiety - depressive, disorders. The symptoms occured during menopause, after loss of life-partner (separation or death). It was not estabished, which of these disorders appeared as the first one. The prognosis was better for the second group of those patients.

## Remschmidt H., Wienand F., & Wewetzer C. (1988). Long term prognosis in anorexia nervosa. Monatsschrift fur Kinderheilkunde, 136(11), 726–731.

Follow-up studies using the same prognostic criteria have shown that about 48% of the patients had recovered at follow-up, while 28% revealed further difficulties with eating, weight and figure, and 24% remained anorectic. Our own study on 103 patients who underwent inpatient treatment, revealed unexpectedly good results: according to the criteria of Morgan and Russell, 72% (n = 58) showed a good prognosis, 11% (n = 9) a fair, and 17% (n = 14) an unfavourable prognosis. 3 patients had died during the follow-up interval. It was possible to predict the long-term outcome from weight recovery during inpatient treatment, more successfully in patients with favourable than unfavourable outcome. The best predictors were: time until weight stabilization, the ratio ideal weight/stabilized weight, and age at onset of the eating disorder.

## Riku K. (2004). Outcome of outpatients with eating disorders. Journal of the Osaka City Medical Center, 53(1), 17–23.

It is well known that eating disorders develop under the strong influence of socio-cultural background. Psychiatric treatment systems including eating disorder treatments also differ markedly between Japan and Western countries. In addition, outcome studies on bulimia nervosa (BN) patients are scanty compared with those on anorexia nervosa (AN), because the concept of bulimia nervosa has been established for only two decades. The aim of the current study is to demonstrate the outcome of patients with AN or BN receiving outpatient treatment only during more than 6 months at least four years ago. Subjects consisted of 11 patients with anorexia nervosa restricting type (AN-R), 12 patients with anorexia nervosa binge-eating/purging type (AN-BP), and 29 patients with bulimia nervosa (BN). Their diagnoses were based on DSM-F criteria. Approximately 10 years elapsed since their first visit in most of the subjects. Main outcome assessment scale was the Morgan-Russell General Outcome Category. Nine (82%) of AN-R, 6 (50%) of AN-BP, and 19 (66%) of BN improved, and were categorized as good by the General Outcome Category. One patient who was AN-R patient had died. These results suggested that there were no differences between the outcome of Japanese outpatients with AN or BN and those in Western countries.

## Sigurdardottir, A., Palsson, S. P., & Thorsteinsdottir, G. (2010). [Anorexia nervosa in psychiatric units in Iceland 1983-2008, incidence of admissions, psychiatric comorbidities and mortality]. Lystarstol 1938-2008 - Innlagnir, Sjukdomsmynd Og Lifun., 96(12), 747–753. https://doi.org/10.17992/lbl.2010.12.331

OBJECTIVE: Information is scarce concerning the incidence of anorexia nervosa (AN) in psychiatric facilities in Iceland. The aim of this study was to describe the incidence of admissions, comorbidity and mortality of patients who were admitted to psychiatric units in Iceland, diagnosed with AN in 1983-2008., MATERIAL AND METHODS: The study is retrospective. 140 medical records with an AN or atypical eating disorder diagnosis according to the ICD-9 and ICD-10 were reviewed. Final sample was 84 patients with confirmed AN diagnosis., RESULTS: Five men and 79 women were admitted to a psychiatric inpatient ward for the first time diagnosed with AN. Average age was 18.7 years. Incidence of admissions for both sexes in the first part of the study period (1983-1995) was 1.43/100.000 persons/year, 11-46 years old, but in the second part (1996-2008) 2.91. The increase was statistically significant (RR=2.03 95% CI 1.28-3.22) and can mainly be explained by an increased incidence of admissions to the children- and adolescent psychiatric wards (CAW). Mortality of women was 2/79 (2.5%) and standard mortality rate 6.25. The average length of stay was 97 days, 67.3 days in adult units and 129.7 days in CAW (p<0.05). In the study period 51 patients (60.7%) were only admitted once. One patient had compulsory admission on his first admission but ten (11.9%) had at some point compulsory admission. The body mass index increased in average from admission to discharge from 15.3 to 17.5 kg/m2. A correlation was found between self harm and suicide attempts and compulsory admissions., CONCLUSION: The study revealed an increased incidence between periods. This might reflect a real increase of AN in the society. Mortality rate was lower than expected.

## Suokas, J., Gissler, M., Haukka, J., Linna, M., Raevuori, A., & Suvisaari, J. (2015). [Outcome of eating disorder patients treated in tertiary care]. Erityishoidon Yksikossa Hoidettujen Syomishairiopotilaiden Ennuste., 131(8), 744–752.

BACKGROUND: We assessed the outcome of eating disorder patients treated in a specialized treatment setting., MATERIAL AND METHODS: Register-based follow-up study of adults (n = 2 450, 95% women, age range 18-62 years). For each patient four background-matched controls were selected., RESULTS: The hazard ratio for all-cause mortality was 6.51 in anorexia, 2.97 in bulimia and 1.77 in BED. Autoimmune diseases were more common in patients than in controls. Bulimia and BED were associated with increased type 2 diabetes risk. Pregnancy and childbirth rates were lower among patients than among controls., CONCLUSIONS: Eating disorders are associated with multiple health problems and increased mortality risk.

## Viricel, J., Bossu, C., Galusca, B., Kadem, M., Germain, N., Nicolau, A., Millot, L., Vergely, N., Lassandre, S., Carrot, G., Lang, F., & Estour, B. (2005). [Retrospective study of anorexia nervosa: Reduced mortality and stable recovery rates]. Diminution de La Mortalite et Stabilite Du Taux de Guerison Dans Le Suivi de l’anorexie Mentale., 34(20), 1505–1510.

OBJECTIVES: Anorexia nervosa is an eating disorder that combines malnutrition, amenorrhea, and distorted body image. To learn more about the course of this disease we undertook a retrospective study of girls diagnosed with anorexia nervosa in the Saint Etienne Endocrinology Department between 1979 and 2004., METHODS: Patients were diagnosed according to DSMIV criteria. Data collected to complete the Morgan-Russell outcome assessment schedule included chronology of illness, patients’ morphological features, anorexia type, treatment choice, patient’s gynecological history, and social status., RESULTS: The study included 206 cases. The average follow-up period was 8.3 +/- 5.3 years. Defining recovery as stable BMI>17.5 kg/m2 for at least one year and recovery of normal menstruation, full recovery was observed in 55.8% and partial recovery in 25.7%, while 18.5% remained chronically ill. Early onset (i.e., during adolescence) was associated with good prognosis, and advanced emaciation and delayed or insufficient medical care with poor prognosis., CONCLUSIONS: The seriousness of this disease is due more to the incidence of cases that become chronic than to the mortality rate. Prediction of severity would be improved by taking into account underlying personality traits, such as addictive tendencies and depression.

## Willi J., Limacher B., Helbling P., & Nussbaum P. (1989). Ten-year follow-up of anorexia patients first hospitalized in the Canton of Zurich during 1973-1975. Schweizerische Medizinische Wochenschrift, 119(5), 147–155.

Out of all 38 anorexia nervosa patients hospitalized for the first time in a private or state hospital for pediatrics, internal medicine or psychiatry in the Canton of Zurich during the period 1973-1975, 28 cases could be assessed 10 years later using a semistructured interview. Catamnestic data were categorized according to Morgan and Russel (1975) in a general outcome score and an average outcome score. Regarding somatic and psychosocial dimensions, 11% of the patients examined can be considered symptom-free, 71% improved and 14% unchanged. One patient (4%) had died from the sequelae of anorexia. 64% increased in weight, nevertheless more than half of the patients had disturbed eating behavior at the time of this catamnestic study. An equally frequent finding was considerable impairment of psychologic well-being chiefly reflected in anxiety and depression. The time necessary to attain the normal weight range had varied between a few months and 11 years. Of 8 variables often claimed to be prognostic in the literature on the course of anorexia nervosa, only a short duration of illness before hospitalization was shown to be relevant. The patients with good or medium outcome most often consider psychotherapy or a partner relationship a decisive factor in the favorable course of their eating problem.

##

## 8. Outlier and Influence Analyses Figures

**Figure 8a**

*Baujat plot*


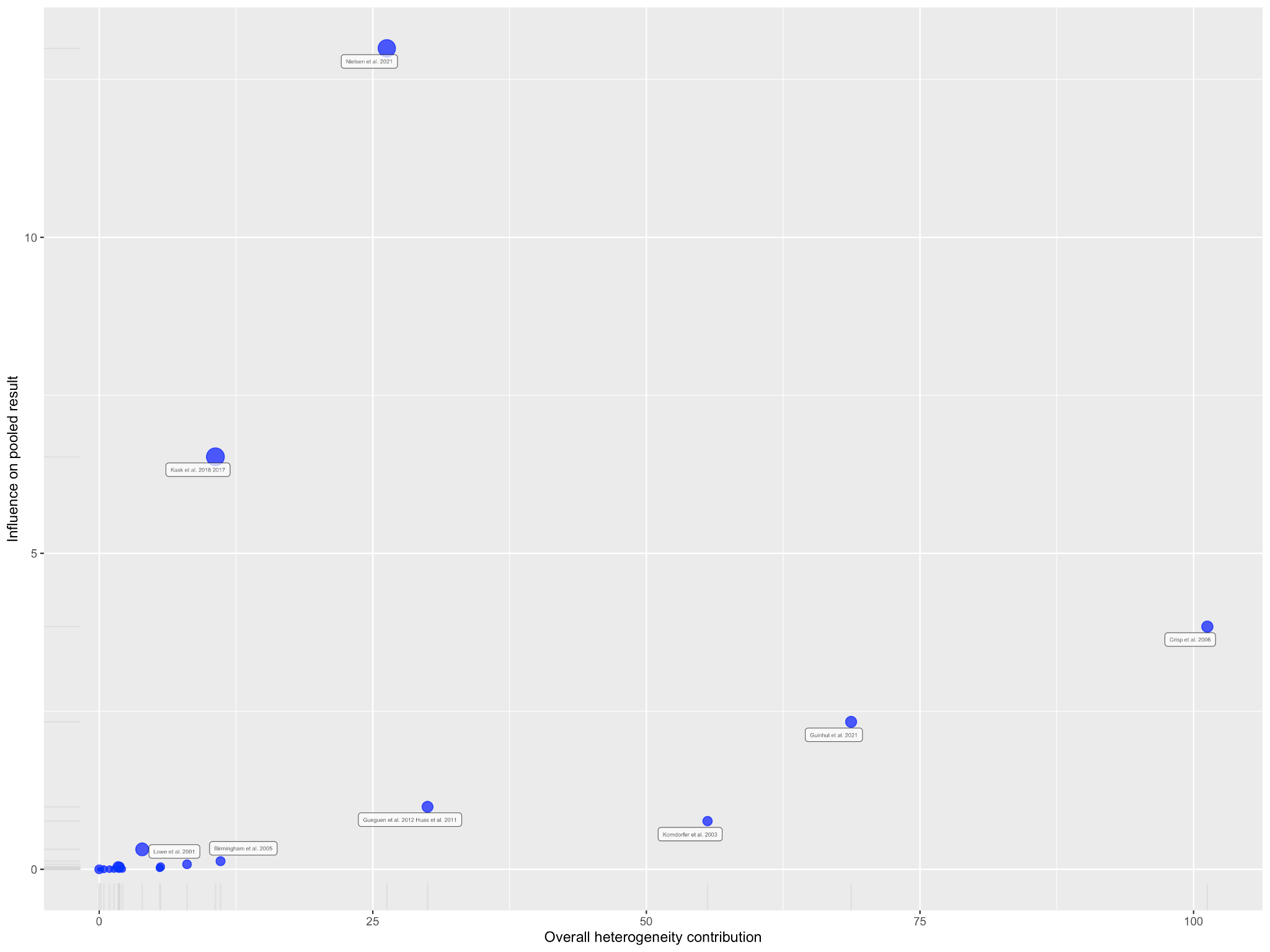


*Note.* Baujat plot showing the contribution of each study to the overall heterogeneity on the x-axis and its influence on the pooled effect size on the y-axis.

**Figure 8b**

*Influence analysis*


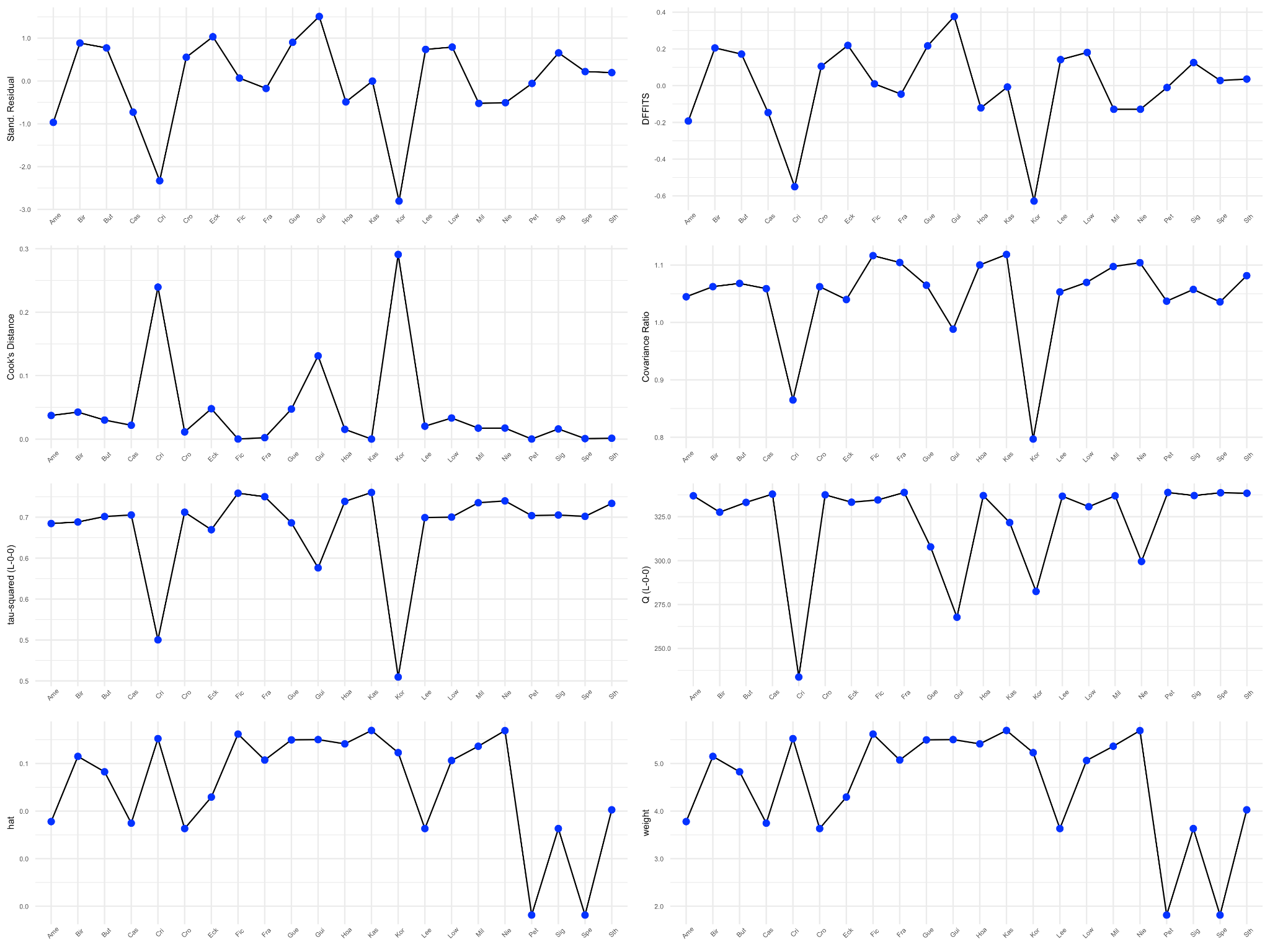


*Note.* No study is identified consistently as an influencer across eight different influence measures.

**Figure 8c**

*Leave-one-out Analysis Ranked by Effect Size*


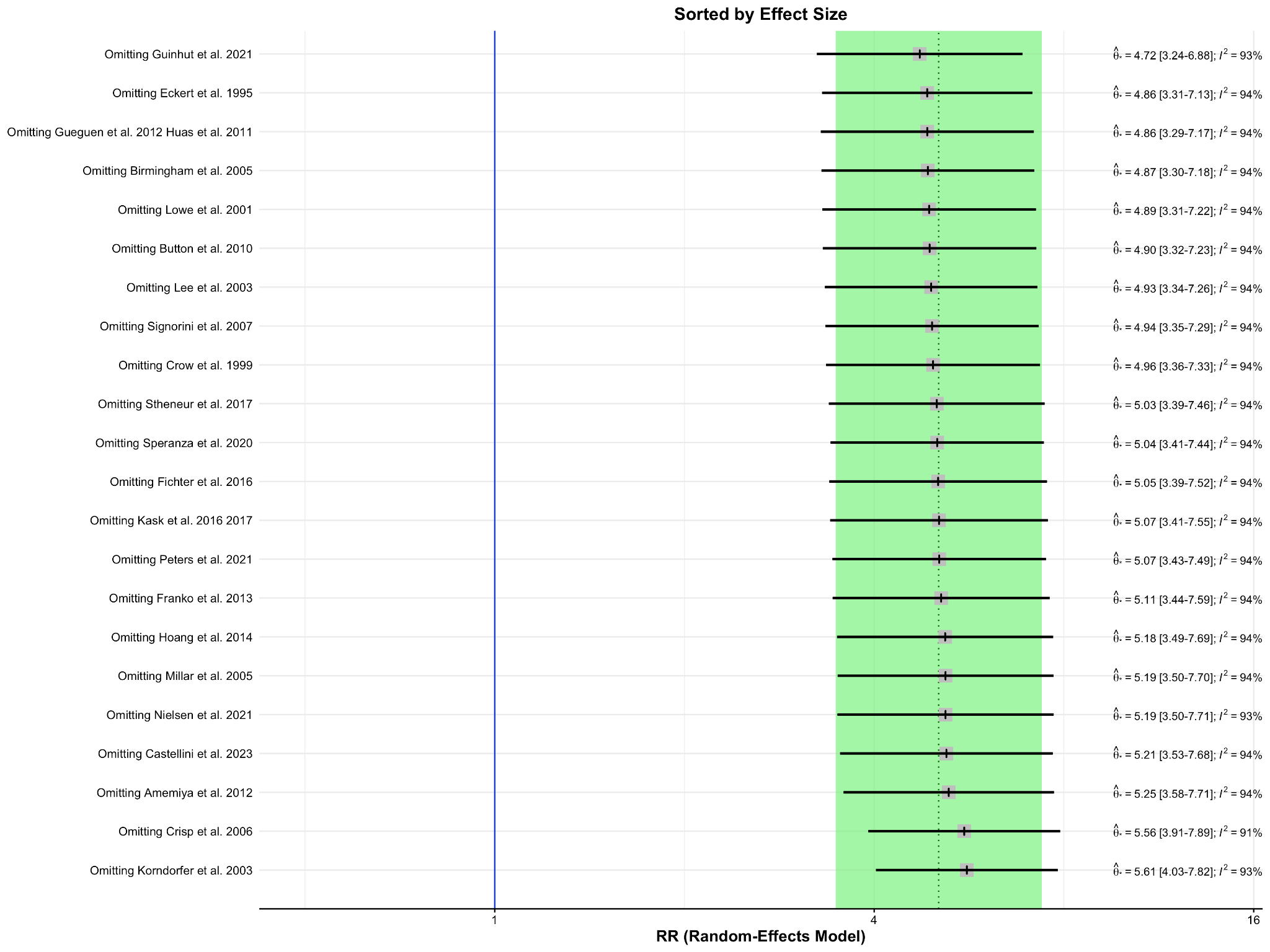


**Figure 8d**

*Leave-one-out Analysis Rank by I^2^*


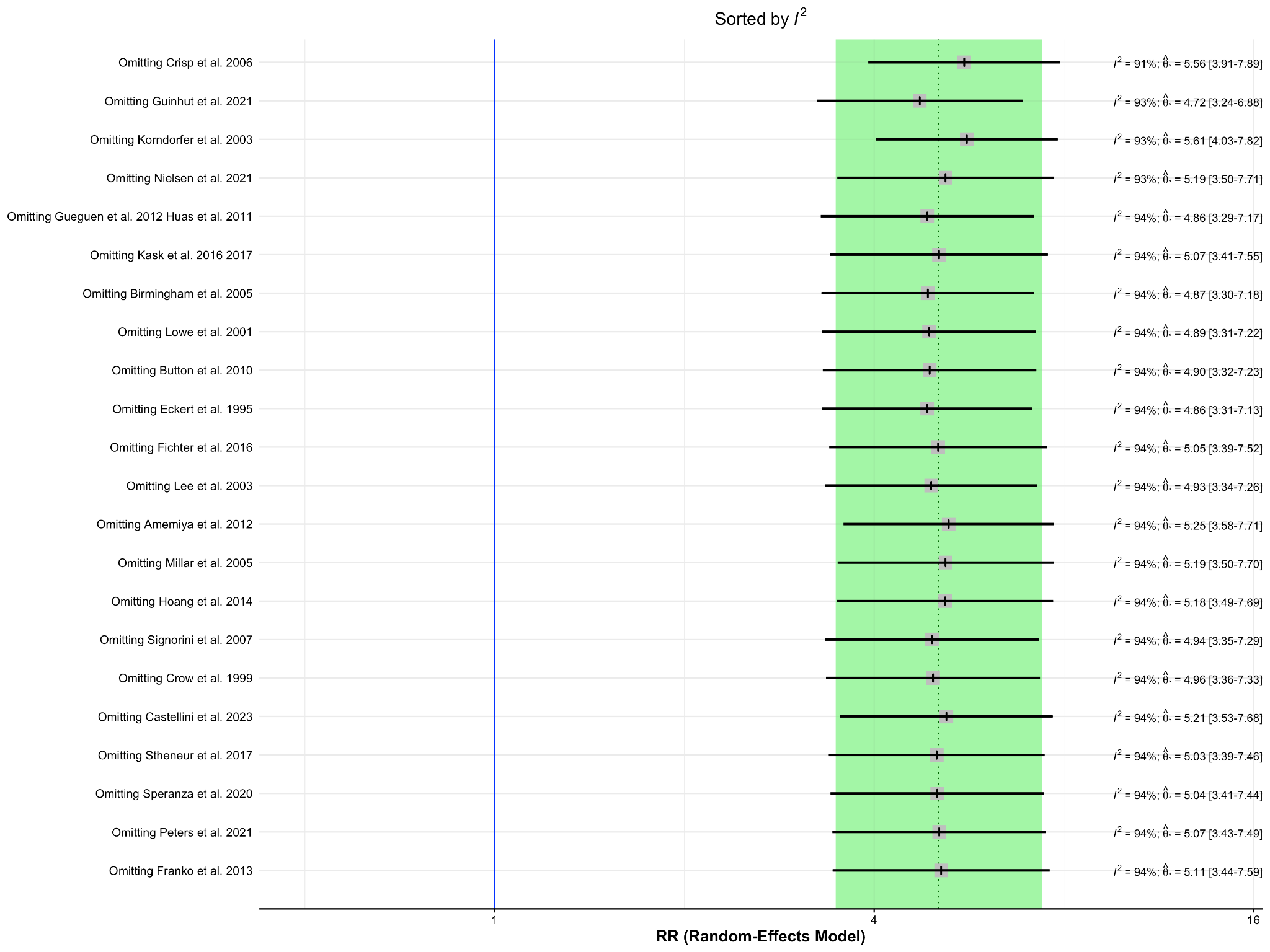


## 9. Sensitivity Analysis Figures

**Figure 9a**

*Forest Plot of the Pooled SMR of All 28 Study Units of Patients with Anorexia Nervosa*


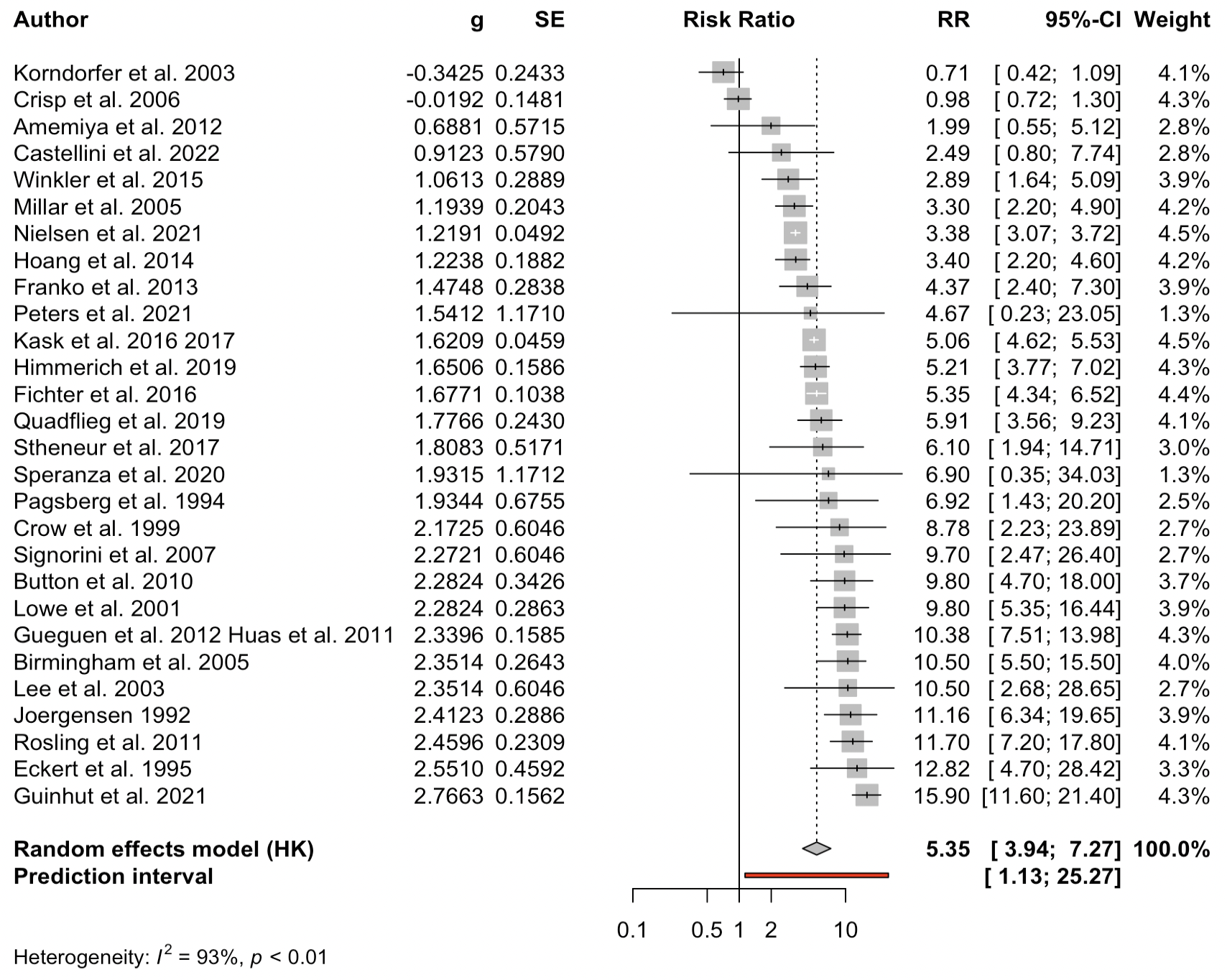


*Note.* Sensitivity analysis was done by including all 28 study units satisfying the inclusion and exclusion criteria. This approach pooled together all eligible data, and partial overlap was allowed.

## 10. Figures for the Analysis of Studies with Male-only Patients Compared with Female Patients

*Power Analysis of Subgroup Analysis of Gender Difference*


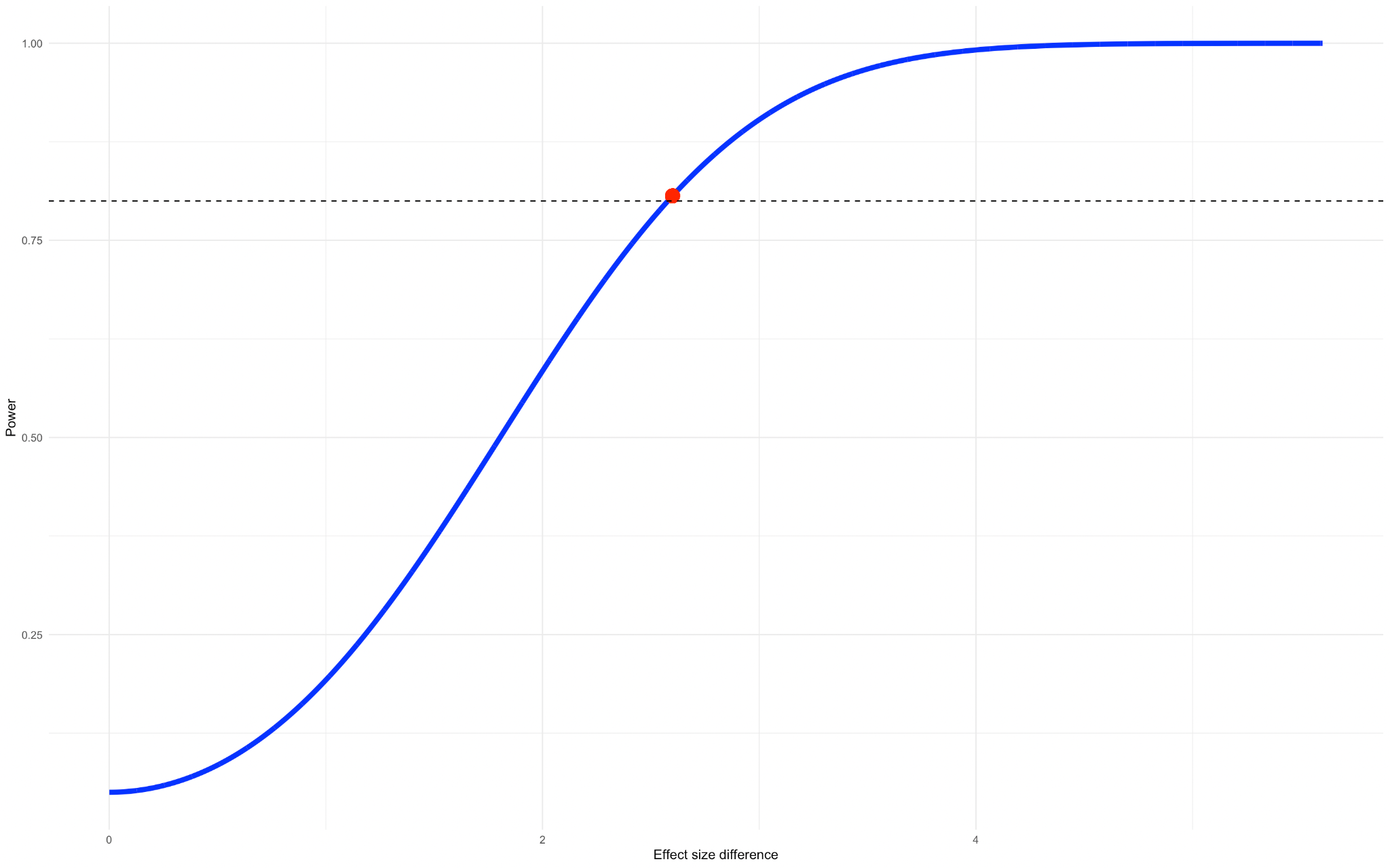


*Note*. This power analysis assumes the expected pooled SMR is 7.2 for male and 4.6 for female studies. The standard deviation is estimated to be 3.9 from the 28 study units in our meta-analysis. This analysis found that we need 35 studies for male and female AN mortality to achieve a power of 80%.

*Funnel Plot for the 9 Male and 9 Female AN Samples For Studying Gender Difference*


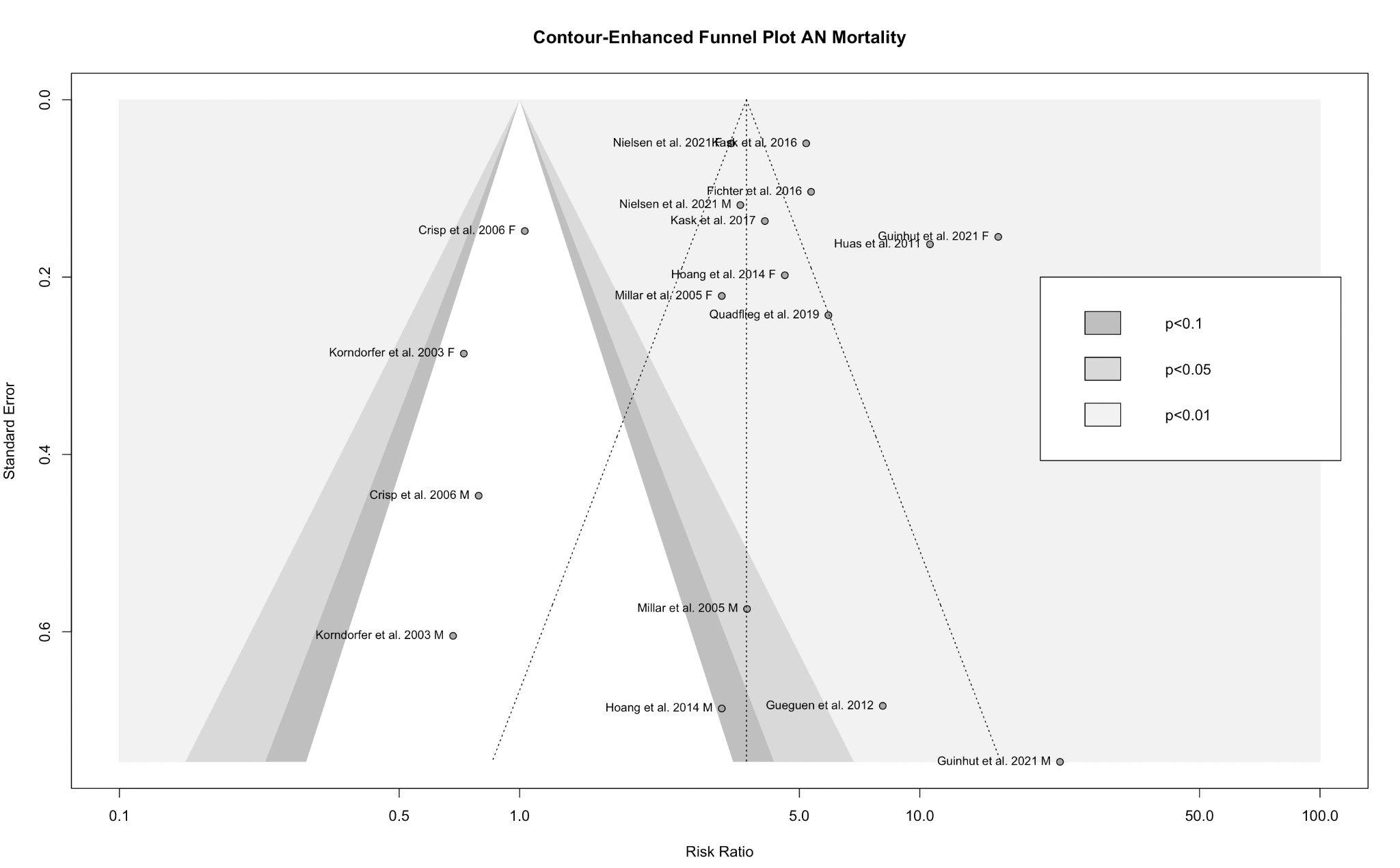


*Note*. The funnel plot for the 9 male and 9 female AN study samples showed no asymmetry.

*Eggers’ test of the intercept showed the intercept to be -0.594, with a 95% CI [-3.64 - 2.46] and p = 0.71, which did not indicate the presence of funnel plot asymmetry.

1. http://www.columbia.edu/~cjd11/charles_dimaggio/DIRE/styled-4/code-12/#continuously-distributed-variables-lehrs-equation [↑](#footnote-ref-0)
